# Supplementary material for: Seroprevalence trends of anti-SARS-CoV-2 antibodies in the adult population of the São Paulo Municipality, Brazil: Results from seven serosurveys from June 2020 to April 2022. The SoroEpi MSP Study
Source: PLoS One. 2024 Aug 26;19(8):e0309441. doi: 10.1371/journal.pone.0309441 (PMC11346932; doi:10.1371/journal.pone.0309441)
Supplement: S1 Table — The SoroEpi MSP Study, Municipality of São Paulo, SP, Brazil, June 2020 to April 2022. (DOCX) [file pone.0309441.s001.docx]

**S1 Table.** Frequency of selected characteristics of the sample population per survey. The SoroEpi MSP Study, Municipality of São Paulo, SP, Brazil, June 2020 to April 2022.

| **Characteristic** | **Survey 1** | | | **Survey 2** | | | **Survey 3** | | | **Survey 4** | | | **Survey 5** | | | **Survey 6** | | | **Survey 7** | | |
| --- | --- | --- | --- | --- | --- | --- | --- | --- | --- | --- | --- | --- | --- | --- | --- | --- | --- | --- | --- | --- | --- |
|  |  |  |  |  |  |  |  |  |  |  |  |  |  |  |  |  |  |  |  |  |  |
|  | **n** | **%^a^** | **%^b^** | **n** | **%^a^** | **%^b^** | **n** | **%^a^** | **%^b^** | **n** | **%^a^** | **%^b^** | **n** | **%^a^** | **%^b^** | **n** | **%^a^** | **%^b^** | **n** | **%^a^** | **%^b^** |
| **Overall** | **1183** | **100.0** | **100.0** | **1470** | **100.0** | **100.0** | **1129** | **100.0** | **100.0** | **1194** | **100.0** | **100.0** | **1187** | **100.0** | **100.0** | **1035** | **100.0** | **100.0** | **936** | **100.0** | **100.0** |
| **Study strata (mean income)** |  |  |  |  |  |  |  |  |  |  |  |  |  |  |  |  |  |  |  |  |  |
| High | 676 | 57.1 | 48.3 | 851 | 57.9 | 48.3 | 544 | 48.2 | 48.3 | 580 | 48.6 | 49.2 | 610 | 51.4 | 49.2 | 415 | 40.1 | 49.2 | 435 | 46.5 | 48.0 |
| Low | 507 | 42.9 | 51.7 | 619 | 42.1 | 51.7 | 585 | 51.8 | 51.7 | 614 | 51.4 | 50.8 | 577 | 48.6 | 50.8 | 620 | 59.9 | 50.8 | 501 | 53.5 | 52.0 |
| **Sex** |  |  |  |  |  |  |  |  |  |  |  |  |  |  |  |  |  |  |  |  |  |
| Male | 521 | 44.0 | 46.6 | 607 | 41.3 | 46.6 | 460 | 40.7 | 46.6 | 483 | 40.5 | 46.6 | 481 | 40.5 | 46.6 | 368 | 35.6 | 46.6 | 303 | 32.4 | 46.7 |
| Female | 662 | 56.0 | 53.4 | 863 | 58.7 | 53.4 | 669 | 59.3 | 53.4 | 711 | 59.6 | 53.4 | 706 | 59.5 | 53.4 | 667 | 64.4 | 53.4 | 633 | 67.6 | 53.3 |
| **Age group (years)** |  |  |  |  |  |  |  |  |  |  |  |  |  |  |  |  |  |  |  |  |  |
| 18 - 39 | 496 | 41.9 | 44.6 | 613 | 41.7 | 44.6 | 459 | 40.7 | 44.6 | 497 | 41.6 | 43.8 | 502 | 42.3 | 43.8 | 414 | 40.0 | 43.8 | 325 | 34.7 | 42.9 |
| 40 - 59 | 435 | 36.8 | 35.2 | 494 | 33.6 | 35.2 | 397 | 35.2 | 35.2 | 425 | 35.6 | 35.5 | 462 | 38.9 | 35.5 | 357 | 34.5 | 35.5 | 360 | 38.5 | 35.8 |
| ≥ 60 | 252 | 21.3 | 20.2 | 363 | 24.7 | 20.2 | 273 | 24.2 | 20.2 | 272 | 22.8 | 20.7 | 223 | 18.8 | 20.7 | 264 | 25.5 | 20.7 | 251 | 26.8 | 21.3 |
| **Self-reported race/skin color** |  |  |  |  |  |  |  |  |  |  |  |  |  |  |  |  |  |  |  |  |  |
| White | 614 | 51.9 | 52.2 | 733 | 49.9 | 48.6 | 556 | 49.3 | 48.5 | 609 | 51.0 | 51.0 | 537 | 45.2 | 44.9 | 406 | 39.2 | 41.9 | 435 | 46.5 | 47.4 |
| Black or mixed (P*ardo*) | 510 | 43.1 | 42.9 | 671 | 45.7 | 47.3 | 530 | 46.9 | 47.3 | 549 | 46.0 | 46.3 | 584 | 49.2 | 49.0 | 599 | 57.9 | 55.6 | 470 | 50.2 | 48.9 |
| Asian or Indigenous | 48 | 4.1 | 4.0 | 49 | 3.3 | 3.1 | 33 | 2.9 | 3.5 | 30 | 2.5 | 2.4 | 58 | 4.9 | 5.5 | 25 | 2.4 | 2.2 | 24 | 2.6 | 2.7 |
| Did not answer | 11 | 0.9 | 0.9 | 17 | 1.2 | 0.9 | 10 | 0.9 | 0.7 | 6 | 0.5 | 0.3 | 8 | 0.7 | 0.5 | 5 | 0.5 | 0.3 | 7 | 0.7 | 1.0 |
| **Schooling (years)** |  |  |  |  |  |  |  |  |  |  |  |  |  |  |  |  |  |  |  |  |  |
| ≤11 | 386 | 32.6 | 32.9 | 518 | 35.2 | 33.8 | 429 | 38.0 | 35.6 | 442 | 37.0 | 34.9 | 415 | 35.0 | 34.6 | 417 | 40.3 | 34.4 | 370 | 39.5 | 35.5 |
| 12 - 15 | 464 | 39.2 | 39.7 | 586 | 39.9 | 41.1 | 426 | 37.7 | 39.1 | 474 | 39.7 | 40.2 | 483 | 40.7 | 39.8 | 416 | 40.2 | 41.8 | 381 | 40.7 | 44.2 |
| ≥16 | 333 | 28.2 | 27.4 | 366 | 24.9 | 25.2 | 274 | 24.3 | 25.3 | 278 | 23.3 | 25.0 | 289 | 24.4 | 25.6 | 202 | 19.5 | 23.7 | 185 | 19.8 | 20.2 |

^a^Unweighted estimates.

^b^Estimates weighted by sampling design with adjustments by census tracts response rates and São Paulo adult population characteristics (2020 population data for Surveys 1 to 3; 2021 for Surveys 4 to 6 and 2022 for Survey 7).
