# Supplementary material for: Seroprevalence trends of anti-SARS-CoV-2 antibodies in the adult population of the São Paulo Municipality, Brazil: Results from seven serosurveys from June 2020 to April 2022. The SoroEpi MSP Study
Source: PLoS One. 2024 Aug 26;19(8):e0309441. doi: 10.1371/journal.pone.0309441 (PMC11346932; doi:10.1371/journal.pone.0309441)
Supplement: S2 Table — The SoroEpi MSP Study, Municipality of São Paulo, SP, Brazil, June 2020 to April 2022. (DOCX) [file pone.0309441.s002.docx]

**S2 Table.** Frequency of adults (and 95% Confidence Interval) by type of anti-SARS-CoV-2 vaccine received and age group in Surveys 5, 6 and 7. The SoroEpi MSP Study, Municipality of São Paulo, SP, Brazil, June 2020 to April 2022.

| Age group (years) | Survey 5 | | | | | | Survey 6 | | | | | | Survey 7 | | | | | |
| --- | --- | --- | --- | --- | --- | --- | --- | --- | --- | --- | --- | --- | --- | --- | --- | --- | --- | --- |
|  | One or more doses of CoronaVac vaccine | | Other vaccine types^a^ | | No vaccine | | One or more doses of CoronaVac vaccine | | Other vaccine types^b^ | | No vaccine | | One or more doses of CoronaVac vaccine | | Other vaccine types^b^ | | No vaccine | |
|  | n | %  (95% CI) | n | % (95% CI) | n | % (95% CI) | n | % (95% CI) | n | % (95% CI) | n | % (95% CI) | n | % (95% CI) | n | % (95% CI) | n | % (95% CI) |
| 18 - 39 | 8 | 1.7  (0.8-3.3) | 7 | 1.2  (0.5-2.5) | 487 | 97.2  (95.3-98.3) | 170 | 40.1  (34.7-45.7) | 221 | 53.9  (47.7-60.0) | 23 | 6.0  (3.8-9.3) | 131 | 39.7  (33.8-45.9) | 187 | 58.3  (52.1-64.2) | 7 | 2.0  (0.8-4.7) |
| 40 - 59 | 15 | 3.0  (1.7-5.0) | 14 | 3.0  (1.8-5.0) | 433 | 94.0  (91.6-95.8) | 54 | 13.7  (10.2-18.1) | 292 | 82.5  (78.1-86.2) | 11 | 3.8  (2.0-6.9) | 65 | 17.3  (12.6-23.3) | 288 | 80.1  (74.2-85.0) | 7 | 2.6  (1.1-5.9) |
| ≥ 60 | 95 | 41.8  (35.1-48.9) | 45 | 20.7  (15.9-26.4) | 83 | 37.5  (31.7-43.6) | 127 | 49.4  (41.3-57.5) | 136 | 50.4  (42.2-58.5) | 1 | 2.6  (0.3-1.8) | 117 | 47.0  (40.6-53.6) | 134 | 53.0  (46.4-59.4) | - | - |
| **Total** | **118** | **10.5**  **(8.5-12.9)** | **66** | **5.9**  **(4.7-7.4)** | **1003** | **83.7**  **(81.1-85.9)** | **351** | **32.7**  **(29.0-36.6)** | **649** | **63.3**  **(59.3-67.2)** | **35** | **4.0**  **(2.8-5.8)** | **313** | **33.2**  **(29.6-37.1)** | **609** | **65.0**  **(61.0-68.8)** | **14** | **1.8**  **(0.9-3.5)** |

^a^AstraZeneca

^b^AstraZeneca, Pfizer, Jansen
