# Supplementary material for: Seroprevalence trends of anti-SARS-CoV-2 antibodies in the adult population of the São Paulo Municipality, Brazil: Results from seven serosurveys from June 2020 to April 2022. The SoroEpi MSP Study
Source: PLoS One. 2024 Aug 26;19(8):e0309441. doi: 10.1371/journal.pone.0309441 (PMC11346932; doi:10.1371/journal.pone.0309441)
Supplement: Attachment — Submitted filename: PONE-D-23-14879_R1.pdf [file pone.0309441.s004.pdf]

PLOS ONE

Seroprevalence trends of anti-SARS-CoV-2 antibodies in the adult population of the São Paulo Municipality, Brazil: Results from seven serosurveys from June 2020 to April 2022. The SoroEpi MSP Study

--Manuscript Draft--

**Manuscript Number:**	PONE-D-23-14879R1
**Article Type:**	Research Article
**Full Title:**	Seroprevalence trends of anti-SARS-CoV-2 antibodies in the adult population of the São Paulo Municipality, Brazil: Results from seven serosurveys from June 2020 to April 2022. The SoroEpi MSP Study
**Short Title:**	Seroprevalence trends of anti-SARS-CoV-2 antibodies in São Paulo, Brazil
**Corresponding Author:**	BEATRIZ HELENA TESS, MD, PhD<br>Universidade de São Paulo Faculdade de Medicina: Universidade de Sao Paulo Faculdade de Medicina<br>São Paulo, São Paulo BRAZIL
**Keywords:**	SARS-CoV-2; COVID-19; Prevalence; serosurvey; trends; household; Adult; general population; Brazil
**Abstract:**	Background: Sequential population-based household serosurveys of SARS-CoV-2 comprising the COVID-19 pre and post-vaccination periods are scarce in Brazil. This study aimed to investigate trends of seropositivity among adults in the municipality of São Paulo, Brazil.Methods: We conducted seven cross-sectional surveys of adult population-representative samples between June 2020 and April 2022. The study design included probabilistic sampling, assessment of the presence of SARS-CoV-2 antibodies with the Roche Elecsys anti-nucleocapsid assay, and statistical adjustments for the population demographics and non-response. The weighted seroprevalences with 95% confidence intervals (CI) were estimated by sex, age group, race, schooling, and mean income study strata. Time trends in seropositivity were assessed using the Joinpoint model. We compared infection-induced seroprevalences with COVID-19 reported cases.Results: 8,134 adults composed the study sample. The overall SARS-CoV-2 seroprevalence increased from 11.4% (95%CI: 9.2-13.6) in June 2020 to 24.9% (95%CI: 21.0-28.7) in January 2021; and from 38.1% (95%CI: 34.3-41.9) in April 2021 to 77.7% (95%CI: 74.4-81.0) in April 2022. The prevalence over time was higher in the subgroups of 18–39-year-old-age and self-reported Black or mixed (pardo). Monthly prevalence rose steeply from January 2021 onwards, particularly among those 60 years or older, who were prioritized for receiving vaccines. The infection-to-case ratios ranged from 8.9 in June 2020 to 4.3 in January 2021.Conclusions: We observed that overall seroprevalence rose significantly over time and with age and race subgroups variations. Increases in the 60 years or older age and the White adults were faster than younger ages and Black or mixed (pardo) race groups after the beginning of the vaccination. Our data may add to the understanding of the complex and changing population dynamics of the SARS-CoV-2 infection, including the impact of vaccination strategies and the modelling of future epidemiological scenarios.
**Order of Authors:**	BEATRIZ HELENA TESS, MD, PhD
	Celina Maria Turchi Martelli, MD, PhD
	Maria Cecília Goi Porto Alves, PhD
	Fanny Cortes, MSc
	Regina Tomie Ivata Bernal, PhD
	Expedito José de Albuquerque Luna, MD, PhD
	Wayner Vieira de Souza, PhD
	Laura da Cunha Rodrigues, MD, PhD
	Celso Francisco Hernandes Granato, MD, PhD

Powered by Editorial Manager® and ProduXion Manager® from Aries Systems Corporation

	Edgar Gil Rizzatti, MD, PhD
	Fernando de Castro Reinach, PhD
	Márcia Cavallari Nunes, PhD
	Maria Carolina Tostes Pintão, MD, PhD
**Response to Reviewers:**	We thank both the academic editor and the reviewer for their valuable comments/suggestions. We have revised the entire text according to those comments as presented below.<br><br>Journal Requirements:<br>1.Please ensure that your manuscript meets PLOS ONE's style requirements, including those for file naming.<br><br>Response: We have adjusted our submitting materials to meet the PLOS ONE style templates.<br><br>2.Please include your updated Competing Interests statement in your cover letter; we will change the online submission form on your behalf.<br>Response: We have included the updated Competing Interests statement in our cover letter.<br>3.Please include your full ethics statement in the 'Methods' section of your manuscript file. In your statement, please include the full name of the IRB or ethics committee who approved or waived your study, as well as whether or not you obtained informed written or verbal consent. If consent was waived for your study, please include this information in your statement as well.<br>Response: We have included the information requested by the academic editor in the text (lines 174-178). Please refer to line 191 of the revised manuscript where we stated that "After participants signed the written informed consent, face-to-face interviews were conducted….".<br>4.We note that you have stated that you will provide repository information for your data at acceptance. Should your manuscript be accepted for publication, we will hold it until you provide the relevant accession numbers or DOIs necessary to access your data. If you wish to make changes to your Data Availability statement, please describe these changes in your cover letter and we will update your Data Availability statement to reflect the information you provide.<br>Response: Our dataset can be accessed at the University of São Paulo repository: https://sites.usp.br/epi-di/pagina-1/pagina-1-1/<br><br>Editor's and reviewer's comments:<br>5.The authors should consider several important studies that have looked at (pre-vaccination) SARS-CoV-2 antibodies in Sao Paulo. Namely, the authors need to compare their findings to the studies from Buss et al Science 2021 and Prete et al. eLife 2022, which looked at SARS-CoV-2 antibodies in (more cost effective) blood donor populations in Sao Paulo city using Abbot assays.<br>Response: We have included both references in the text (lines 382 and 390, references number 26 and 27).<br>6.Another important limitation is the lack of adjustments regarding sensitivity and specificity and seroreversion (if any) for the Roche assay.<br>Response: We have added in the text (lines 214-216) the 95%CI for sensitivity (99.5% [95% CI: 97.0-100.0]) and specificity (99.8% [95% CI: 99.7-99.9]) according to the development and validation of the Elecsys Anti-SARS-CoV-2 Immunoassay (Muench et al, 2020). Considering these parameters, the adjustment in prevalence would not produce statistically significant difference in results. In addition, Roche Elecsys is less prone to antibody waning (Muecksch 2021). Furthermore, the seroprevalence trend was not affected since we applied the same assays throughout study period. The study design consisted of seven independent cross-sectional population-based surveys that does not allow to measure antibody waning. Nevertheless, we considered these issues in the discussion section (lines 481-484).<br><br>Minor edits:<br>7.Results section could be restructured to present the survey results in chronological order (e..g reorder lines 282-288).<br>Response: We have made the changes to follow the chronological order.

Powered by Editorial Manager® and ProduXion Manager® from Aries Systems Corporation

	8.Table 2, interpretation would be made easier if all categories were grouped Jun 2020 - Jan 2021; and Jan 2021 - Apr 2022.<br>Response: The time series analysis used in the present study presents parameters and statistical results of the JoinPoint models for each variable. These models identified the number of turning points and the period (trend segments) for each variable. The results of these analyses were able to show distinct growth trends for different categories. In fact, the JointPoint analysis allows us to show changes in the seroprevalence trend. Grouping or fixing the periods is not suitable for time series analysis. For example, the growth model showed no turning point and only one period (straight line for the entire period Jun 2020 - Apr 2022) for the high-income strata. In contrast, the model identified one turning point for the low-income strata and two trend periods (June 2020-October 2020; October 2020-April 2022). In order to make the interpretation easier, we modified the statistical analysis (lines 246-258) and the included a footnote for period in Table 2 (lines 343-344).<br><br>9.Lines 84-85, Sao Paulo was perhaps the most important site of entry of SARS-CoV-2 in the country in the early phase of the pandemic as the authors mention. This has been best shown in Candido et al. JTM 2020 and Candido et al. Science 2021.<br>Response: We have included the two studies by Candido et al in the Introduction section (references number 7 and 8, line 85).<br><br>10. Lines 367-369. Include 95% CIs.<br>Response: We have included the 95% CI in line 369.<br><br>11. Lines 71. Compare and discuss results obtained from seroprevalence studies from Sao Paulo, including Buss et al. Science 2021 and Prete et al. eLife 2022.<br>Response: We have included a paragraph (lines 380-390, references number 26 and 27) in the Discussion section as suggested.
Additional Information:	
Question	Response
**Financial Disclosure**<br><br>Enter a financial disclosure statement that describes the sources of funding for the work included in this submission. Review the submission guidelines for detailed requirements. View published research articles from *PLOS ONE* for specific examples.<br><br>This statement is required for submission and **will appear in the published article** if the submission is accepted. Please make sure it is accurate.	The SoroEpi MSP Study was funded by the Instituto Semeia, Todos pela Saúde, The Grupo Fleury and IBOPE/IPEC. The current analysis did not receive specific funding. Instituto Semeia and Todos pela Saúde had no role in the study concept and design, data collection, analysis, interpretation, or writing of the manuscript. Grupo Fleury and IBOPE contributed to the funding of the SoroEpi MSP by providing their services at or below cost.<br>CMTM received a scholarship (CNPq-Pq): 308974/2018-2; WVS CNPq-Pq: 308000/2021-8.

**Unfunded studies**<br>Enter: *The author(s) received no specific funding for this work.*<br><br>**Funded studies**<br>Enter a statement with the following details:<br>• Initials of the authors who received each award<br>• Grant numbers awarded to each author<br>• The full name of each funder<br>• URL of each funder website<br>• Did the sponsors or funders play any role in the study design, data collection and analysis, decision to publish, or preparation of the manuscript?<br>• **NO** - Include this sentence at the end of your statement: *The funders had no role in study design, data collection and analysis, decision to publish, or preparation of the manuscript.*<br>• **YES** - Specify the role(s) played.<br><br>* typeset	
**Competing Interests**<br><br>Use the instructions below to enter a competing interest statement for this submission. On behalf of all authors, disclose any competing interests that could be perceived to bias this work—acknowledging all financial support and any other relevant financial or non-financial competing interests.<br><br>This statement is required for submission and **will appear in the published article** if the submission is accepted. Please make sure it is accurate and that any funding sources listed in your Funding Information later in the submission form are also declared in your Financial Disclosure statement.<br><br>View published research articles from *PLOS ONE* for specific examples.	BHT, CMTM, MCGPA, FC, RTIB, EJAL, WVS, LCR, and FCR declare no conflict of interest. Authors associated with Grupo Fleury (CFHG, MCTP and EGR) and Ibope Inteligência (MCN) disclose the following potential conflict of interest: the two organizations co-funded the SoroEpi MSP Study by providing their services at or below cost. These include data and blood sample collection and laboratory tests. The companies sell these services in the market and might profit from the publicity generated by the results of this research. This does not alter our adherence to PLOS ONE policies on sharing data and materials.

**NO authors have competing interests**<br><br>Enter: *The authors have declared that no competing interests exist.*<br><br>**Authors with competing interests**<br><br>Enter competing interest details beginning with this statement:<br><br>*I have read the journal's policy and the authors of this manuscript have the following competing interests: [insert competing interests here]*<br><br>* typeset	
**Ethics Statement**<br><br>Enter an ethics statement for this submission. This statement is required if the study involved:<br><br>• Human participants<br>• Human specimens or tissue<br>• Vertebrate animals or cephalopods<br>• Vertebrate embryos or tissues<br>• Field research<br><br>Write "N/A" if the submission does not require an ethics statement.<br><br>General guidance is provided below. Consult the submission guidelines for detailed instructions. **Make sure that all information entered here is included in the Methods section of the manuscript.**	Participants gave written informed consent before data collection. The National Ethics Commission approved the SoroEpi MSP Study under CAAE number 31032620.0.0000.5474 on 14 January 2020, and the Fleury Ethics Committee approved it under the same CAAE number on 12 June 2020.<br>The data were analyzed together and coded for the participants to remain anonymous.

Powered by Editorial Manager® and ProduXion Manager® from Aries Systems Corporation

**Format for specific study types**<br><br>**Human Subject Research (involving human participants and/or tissue)**<br>• Give the name of the institutional review board or ethics committee that approved the study<br>• Include the approval number and/or a statement indicating approval of this research<br>• Indicate the form of consent obtained (written/oral) or the reason that consent was not obtained (e.g. the data were analyzed anonymously)<br><br>**Animal Research (involving vertebrate animals, embryos or tissues)**<br>• Provide the name of the Institutional Animal Care and Use Committee (IACUC) or other relevant ethics board that reviewed the study protocol, and indicate whether they approved this research or granted a formal waiver of ethical approval<br>• Include an approval number if one was obtained<br>• If the study involved *non-human primates*, add *additional details* about animal welfare and steps taken to ameliorate suffering<br>• If anesthesia, euthanasia, or any kind of animal sacrifice is part of the study, include briefly which substances and/or methods were applied<br><br>**Field Research**<br><br>Include the following details if this study involves the collection of plant, animal, or other materials from a natural setting:<br>• Field permit number<br>• Name of the institution or relevant body that granted permission	
**Data Availability**<br><br>Authors are required to make all data underlying the findings described fully available, without restriction, and from the time of publication. PLOS allows rare exceptions to address legal and ethical concerns. See the PLOS Data Policy and FAQ for detailed information.	Yes - all data are fully available without restriction

A Data Availability Statement describing where the data can be found is required at submission. Your answers to this question constitute the Data Availability Statement and **will be published in the article**, if accepted.<br><br>**Important:** Stating 'data available on request from the author' is not sufficient. If your data are only available upon request, select 'No' for the first question and explain your exceptional situation in the text box.<br><br>Do the authors confirm that all data underlying the findings described in their manuscript are fully available without restriction?	
**Describe where the data may be found in full sentences. If you are copying our sample text, replace any instances of XXX with the appropriate details.**<br><br>• If the data are **held or will be held in a public repository**, include URLs, accession numbers or DOIs. If this information will only be available after acceptance, indicate this by ticking the box below. For example: *All XXX files are available from the XXX database (accession number(s) XXX, XXX.*).<br>• If the data are all contained **within the manuscript and/or Supporting Information files**, enter the following: *All relevant data are within the manuscript and its Supporting Information files.*<br>• If neither of these applies but you are able to provide **details of access elsewhere**, with or without limitations, please do so. For example:<br><br>*Data cannot be shared publicly because of [XXX]. Data are available from the XXX Institutional Data Access / Ethics Committee (contact via XXX) for researchers who meet the criteria for access to confidential data.*<br><br>*The data underlying the results presented in the study are available from (include the name of the third party*	All relevant data are within the manuscript, all files are available from the SoroEpi-MSP.xlsx database and are acessible at: https://sites.usp.br/epi-di/pagina-1/pagina-1-1/

and contact information or URL).<br>• This text is appropriate if the data are owned by a third party and authors do not have permission to share the data.<br><br>* typeset	
Additional data availability information:	

Powered by Editorial Manager® and ProduXion Manager® from Aries Systems Corporation

Manuscript clean Click here to access/download;Manuscript;Manusc 23-14879 revised no marks.docx

Seroprevalence trends of anti-SARS-CoV-2 antibodies in the adult population of the São Paulo Municipality, Brazil: Results from seven serosurveys from June 2020 to April 2022. The SoroEpi MSP Study

Beatriz Helena Tess[1*¶^], Celina Maria Turchi Martelli[2¶], Maria Cecília Goi Porto Alves[3^], Fanny Cortes[4], Regina Tomie Ivata Bernal[5], Wayner Vieira de Souza[2], Expedito José de Albuquerque Luna[1], Laura da Cunha Rodrigues[6], Marcia Cavallari Nunes[7^], Fernando de Castro Reinach[8^], Celso Francisco Hernandes Granato[9^], Edgar Gil Rizzatti[9^], Maria Carolina Tostes Pintão[9^]

[1]Departamento de Medicina Preventiva, Faculdade de Medicina, Universidade de São Paulo, São Paulo, SP, Brasil

[2]Instituto Aggeu Magalhães, Fiocruz, Recife, PE, Brasil

[3]Instituto de Saúde, Secretaria de Saúde do Estado de São Paulo, São Paulo, SP, Brasil

[4]Instituto de Avaliação de Tecnologia em Saúde, Porto Alegre, RS, Brasil

[5]Programa de Pós-Graduação, Escola de Enfermagem, Universidade Federal de Minas Gerais, Belo Horizonte, MG, Brasil

[6]Department of Infectious Disease Epidemiology, London School of Hygiene & Tropical Medicine, London, UK

[7]Inteligência em Pesquisa e Consultoria (IPEC), São Paulo, SP, Brasil

[8]Departamento de Bioquímica, Instituto de Química, Universidade de São Paulo, São Paulo, SP, Brasil

[9]Divisão de Pesquisa e Desenvolvimento, Grupo Fleury, São Paulo, SP, Brasil

*Corresponding author

E-mail: beatriz.tess@usp.br (BHT)

¶These authors contributed equally to this work.

^Members of the SoroEpi MSP Study Research Group.

Abstract

Background: Sequential population-based household serosurveys of SARS-CoV-2 covering the COVID-19 pre- and post-vaccination periods are scarce in Brazil. This study aimed to investigate trends of seropositivity among adults in the municipality of São Paulo, Brazil.

Methods: We conducted seven cross-sectional surveys of adult population-representative samples between June 2020 and April 2022. The study design included probabilistic sampling, assessment of the presence of SARS-CoV-2 antibodies using the Roche Elecsys anti-nucleocapsid assay, and statistical adjustments for population demographics and non-response. The weighted seroprevalences with 95% confidence intervals (CI) were estimated by sex, age group, race, schooling, and mean income study strata. Time trends in seropositivity were assessed using the Joinpoint model. We compared infection-induced seroprevalences with COVID-19 reported cases.

Results: The study sample comprised 8,134 adults. The overall SARS-CoV-2 seroprevalence increased from 11.4% (95%CI: 9.2-13.6) in June 2020 to 24.9% (95%CI: 21.0-28.7) in January 2021; and from 38.1% (95%CI: 34.3-41.9) in April 2021 to 77.7% (95%CI: 74.4-81.0) in April 2022. The prevalence over time was higher in the subgroups 18–39-years of age and self-reported Black or mixed (*Pardo*) race. Monthly prevalence rose steeply from January 2021 onwards, particularly among those aged 60 years or older, who were prioritized for vaccination. The infection-to-case ratios ranged from 8.9 in June 2020 to 4.3 in January 2021.

Conclusions: We observed that the overall seroprevalence rose significantly over time and with age and race subgroup variations. Increases in the 60 years or older age and the White adult groups after the beginning of the vaccination campaign were faster than in younger ages and Black or mixed (*Pardo*) race groups. Our data may add to the understanding of the complex and changing population dynamics of the SARS-CoV-2 infection, including the impact of vaccination strategies and the modelling of future epidemiological scenarios.

Keywords

SARS-CoV-2, COVID-19, prevalence, serosurvey, adult, general population, Brazil

Introduction

Brazil was one of the countries most affected by the COVID-19 pandemic [1]. From February 2020 to 20 September 2022, approximately 34.6 million confirmed cases of COVID-19, with 685,000 deaths, were reported [2]. During that period, Brazil contributed to 5.6% of the global COVID-19 cases and around 10% of the total reported deaths [3], even though it accounts for only 2.3% of the world population [4].

São Paulo is among the top ten metropolitan areas of the world. It is in the wealthiest Brazilian region and has a high level of socioeconomic inequality [5]. The city of São Paulo, with a population of approximately 11.9 million [6], was the site of SARS-CoV-2 entry into the country, the initial epicenter of the pandemic, and an important route of viral spread [7,8]. As of 14 September 2022, the São Paulo Municipality reported 2,281,679 confirmed cases and 43,785 deaths [9].

Cross-sectional surveys are snapshots of infection history. They can complement data based on PCR-confirmed cases by identifying asymptomatic infections and the magnitude of transmission in different settings and populations [10]. Repeated serosurveys can monitor the spread of the virus in the general population and describe the geographical and subgroup changes over time. Knowledge of trends of SARS-CoV-2 infections can inform and help evaluate the impact of public health actions to control the pandemic and shed light on the dynamics of the interaction between the virus and the population [11].

A systematic review of global SARS-CoV-2 seroprevalence from January 2020 to May 2022 indicated that most population-based studies were conducted

before mass COVID-19 vaccination, and very few studies sampled populations in 2022 [12]. In Brazil, a nationwide household survey showed city-level prevalence had increased from 0% to 25.4% by June 2020, with a high heterogeneity among regions [13]. In the municipality of São Paulo, as part of the local public health response, sequential population-based household surveys estimated 9.7% SARS-CoV-2 seroprevalence in July 2020, with an increase to 25% by February 2021 [14]. SARS-CoV-2 had been difficult to control and still imposes challenging uncertainties, and serial population-based studies help understand the infection's complex and changing population dynamics.

The objectives of this study were to describe the SARS-CoV-2 seroprevalence estimates in the municipality of São Paulo and to explore the seroprevalence trends by sociodemographic characteristics, considering pre- and post-vaccine implementation in the city. Lastly, we compared the overall seropositivity in the adult population with the COVID-19 cumulative registered cases during the pre-vaccination period.

Materials and Methods

Study design and sampling

The SoroEpi Study in the municipality of São Paulo (SoroEpi MSP) comprised seven household-SARS-CoV-2 serosurveys from June 2020 to April 2022: Survey 1 (15-24 June 2020), Survey 2 (20-29 July 2020), Survey 3 (1-10 October 2020), Survey 4 (14-23 January 2021), Survey 5 (22 April-1 May 2021), Survey 6 (9-10 September 2021), and Survey 7 (31 Mar-9 April 2022). Fig 1 shows the number of households and adults by survey, which followed the study design.

Fig 1. Flow diagram for recruitment into the study by serosurvey, The SoroEpi MSP, Municipality of São Paulo, SP, Brazil, June 2020 to April 2022

Before initiating the full-scale surveys, a pilot study was conducted in May 2020, restricted to the six most COVID-19-affected districts in São Paulo. The data collection procedures proved to be adequate and feasible. The detailed methodology and preliminary seroprevalence of SARS-CoV-2 were published previously [15]. To provide the COVID-19 local epidemiological context for each survey time frame, secondary data were retrieved from the official sources of the reported cumulative cases and deaths (Fig 2). We also retrieved SARS-CoV-2 variants circulating during the period of surveys in São Paulo state, traced by GISAID [16]. From June 2020 to January 2021 the predominant circulating variant

was B1.1.28 with the entry of the Gamma variant of concern (VOC) at the beginning of 2021. The Gamma variant was the main circulating VOC by April 2021; Delta VOC by September 2021 and Omicron VOC by April 2022.

Fig 2. Timeline of COVID-19 cumulative cases and deaths according to seroprevalence surveys periods, The SoroEpi MSP Study, Municipality of São Paulo, Brazil, 2020-2022.

The surveys were designed following the World Health Organization (WHO) protocol for population-level COVID-19 antibody testing [17]. They were cross-sectional population-based studies, and the target population was the adult (18 years old or older) inhabitants of permanent private households in the municipality of São Paulo.

According to the 2020 projections for the municipality of São Paulo by the Statewide System for Data Analysis Foundation (Fundação SEADE), 9,164,926 residents were 18 years of age and over out of an estimated total population of 11,869,660 [18]. Administratively, the municipality is organized into 96 districts. For this study, the adult population was divided into two strata of equal size using the average income of the municipality districts as the stratification variable. A sample size of 560 individuals was planned in each stratum to allow the estimation of seroprevalence greater than 4% to be obtained under coefficients of variation of less than 30%, considering a design effect of 2 (deff = 2).

We used a two-cluster probability sampling method to select the survey participants. In the first stage, we selected census tracts (115 in Survey 1 and 160 in the other six surveys) using probability proportional to the number of households counted in the 2010 census. In the second stage, we randomly selected households from each tract (12 in Survey 1 and eight in the subsequent surveys) from a list of households obtained by plotting out the tract. All adults in the selected households were eligible to participate in the study, except those with cognitive incapacities or health problems that make venipuncture difficult. Weights were introduced in the analysis to correct the different probabilities with which participants were randomly selected. These design weights were adjusted for non-response considering the response rates of three sets of census tracts (ordered by average income, according to census data) and for the age and sex distribution observed in the city of São Paulo in the years 2020, 2021, and 2022.

Ethics and study procedures

Approval for the study protocol was obtained from the National Research Ethics Commission (CONEP) and the Research Ethics Committee of Fleury Group (CAAE no 31032620.0.0000.5474). An amendment to the original serology survey was also approved by the Brazilian National Research Ethics Commission (CONEP) (CAAE no 31032620.0.0000.5474 v5) on January 14, 2021.

Three visits were scheduled for each household on different days of the week and times of the day to limit non-response. The sampling method did not permit replacements of the randomly selected sampling units, i.e., the census tracts and households. We recorded the frequency of households with eligible

adults and the frequency of individuals interviewed with valid serological samples in each survey. We estimated the overall participation rate considering the number of included participants divided by the total number of individuals who would have been included according to the sampling frame, but who were considered non-participants because they were not reachable, or households could not be accessed. We assumed that the number of individuals per household was similar between the included households and the non-accessible selected households.

After participants signed the written informed consent, face-to-face interviews were conducted using a questionnaire on mobile devices to obtain socio-demographic and other data. The information used in the present study comprised: sex, age, skin color/race and education measured by years of formal schooling. Self-reported skin color/race categories followed the Brazilian Institute of Geography and Statistics (IBGE) classification: White, Black, mixed race (*Pardo*), Asian, and Indigenous [19]. Questions about the date of vaccination, the number of doses, and the type of vaccine product were included in the questionnaire to account for the introduction of vaccination during Surveys 5, 6, and 7. The COVID-19 immunization program commenced nationally on 17 January 2021, and on the same date in São Paulo city (Fig 2). Four vaccines have been offered in the country's immunization programs: CoronaVac (Sinovac), an inactivated whole-virus COVID-19 vaccine, ChAdOx1 (AstraZeneca), Ad26.COV2.S (Janssen), and BNT162b2 (Pfizer-BioNTech).

Following the interview, about 3-5 ml of venous blood was collected from each participant, and samples were transported to the reference laboratory for

antibody testing. The laboratory team had access to information on individual participants during and after data collection to enable them to provide details of their results and any additional information the participants needed.

Serology testing

Specimens were tested for anti-nucleocapsid total antibodies using the electrochemiluminescence Roche Elecsys Total Ig Assay (Roche Elecsys® Diagnostics, Rotkreuz, Switzerland) in Surveys 2 to 7. The sensitivity of the Elecsys Anti-SARS-CoV-2 immunoassay is 99.5% (95% CI 97-100) and specificity 99.8% (95% CI 99.7-99.9), making it a useful tool for determining past SARS-CoV-2 infection, including at population level [20]. For Survey 1, the specimens were tested for immunoglobulin (Ig) M (IgM) and IgG using Maglumi SARS-CoV-2 chemiluminescence assay Immunoglobulin (Snibe Diagnostics, Shenzhen, China) [21] since that was the available test in the early months of the COVID-19 pandemic in 2020. ANVISA, the Brazilian Regulatory Health Agency, has cleared both assays for emergency use. Other tests were used in parallel with the Elecsys assay during the study period. However, for the sake of the present analyses, we opted to measure the anti-nucleocapsid total antibodies using Roche Elecsys due to its reported high sensitivity and specificity and because it has been broadly used in seroprevalence studies [20,22]. All tests were performed following the manufacturer’s guidelines in the Grupo Fleury laboratory in São Paulo, Brazil.

Primary outcome

The primary outcome was seropositivity, which was defined according to previously set laboratory parameters. Individuals whose sera were reactive to the Maglumi assay in Survey 1 or Roche assay in Surveys 2 to 7 were considered positive. Indeterminate results were included as negative in the analyses. After the CoronaVac vaccine was introduced in early 2021, seropositivity could be determined either by a viral infection or by vaccination and herein is defined as “hybrid immunity” [23].

Statistical analysis

Unadjusted and weighted frequency distributions were calculated with 95% confidence intervals (CI) by sex, age group, self-reported skin color, schooling, and mean income study design strata. Differences between categories were assessed using Rao-Scott chi-square tests. Estimates were obtained using the survey module of STATA version 14 (StataCorp, College Station, Texas, USA), in which the complex aspects of the sampling design were considered.

We applied joinpoint regression analysis to study the trends of sequential seroprevalence estimates of SARS-CoV-2 antibody surveys from June 2020 to April 2022 (seven surveys) using the JoinPoint app [24]. This model allowed us to test whether the changes in the seroprevalence trend were statistically significant and to identify the time of the turning point separating the trend into distinct segments. Our dataset allowed us to choose one as the maximum number of joinpoints in the linear model $y = \alpha + \beta x$. The model selection used

Monte Carlo permutation tests to determine the optimal number of joinpoints [25]. Each one performs a test of the null hypothesis H_0: 0 joinpoint against the alternative H_a: 1 joinpoint. This time series approach can distinguish different growth trends and their period (trend segments) according to the variables analysed. Therefore, the location of the joinpoint (period) was based on statistical criteria resulting from the joinpoint calculations.

Calculation of the reported fraction

We calculated the reported fraction as the ratio of the estimated number of infected individuals using primary seroprevalence data (Surveys 1 to 4) and the number of cumulative cases of COVID-19 extracted from the São Paulo Municipal Surveillance System. We considered the median time of the corresponding fieldwork period. The reported fractions were calculated for the surveys performed before the mass CoronaVac vaccination was introduced in São Paulo.

Results

For each survey, Fig 1 shows the number of randomly selected households (total n = 8,748) and the number of households identified with eligible adults (total n = 3,904), after excluding households which were closed during the data collection periods (total n = 1,436) and those that preferred not to give any information (total n = 3,408). Overall, 44.6% of the randomly selected households were included in the study.

The seven consecutive surveys included 8,134 participants with complete interviews and blood specimens, which corresponded to 73.9% of the 11,013 eligible adults. However, if we consider the participation rate of the selected households together with the participation rate of the eligible adults, the overall participation rate was 38.4% (95% CI 37.7-39.1).

For each survey, the percentages of participants by sex, age group, and mean income study design strata agreed with the percentages of the population of the municipality of São Paulo after the sampling design weights were adjusted for these variables. Regarding race, approximately 50% of the participants self-declared as White, followed by approximately 42% as Black or mixed race (*Pardo*), and less than 5% as Asian or Indigenous. The percentage of participants with 11 years or less of schooling ranged from 32.9% (Survey 2) to 39.5% (Survey 7) (S1 Table).

From June 2020 (Survey 1) to January 2021 (Survey 4), before mass vaccination, the weighted overall seroprevalence increased from 11.4% (95% CI: 9.2-13.6) to 24.9% (95% CI: 21.0-28.7). From April 2021 (Survey 5) to April 2022

(Survey 7), the seropositivity increased from 38.1% (95% CI: 34.3-41.9) to 77.7% (95% CI: 74.4-81.0). Residents in the lower-income stratum had a higher weighted prevalence of SARS-CoV-2 antibodies than the high-income stratum in all surveys except for Survey 2. None of the surveys showed a significant difference in the frequency of seropositivity between women and men. Among the oldest age group, the prevalence ranged from 11.1% (95% CI: 6.0-16.0) in Survey 1 to 65.4% (95% CI: 58.1-72.6) in Survey 7, while in the youngest age group, it varied from 10% (95% CI: 7.1-12.8) to 85.8% (95% CI: 81.6-90.0). The self-declared Black or mixed race (*Pardo*) group presented statistically higher seroprevalence in all surveys when compared to the White group. The population with 16 or more years of education had lower seroprevalence, in all except in the last two surveys (Table 1).

In April 2021 (Survey 5), 83.7% (95% CI: 81.1-85.9) of the São Paulo city population were unvaccinated, and 10.5% (95% CI: 8.5-12.9) had received at least one dose of CoronaVac. There was a sharp decline in the percentage of unvaccinated adults to 4.0% (95% CI: 2.8-5.8) in September 2021 (Survey 6) and 1.8% (95% CI: 0.9-3.5) by April 2022 (Survey 7). The CoronaVac vaccine (one or more doses) was the most commonly used in the oldest age group (60 years or older) and the 18-39 years group (S2 Table).

Table 1. Weighted seroprevalence estimates[a] (and 95% CI) of anti-SARS-CoV-2 per survey, The SoroEpi MSP Study, Municipality of São Paulo, SP, Brazil, June 2020 to April 2022.

Characteristic	Survey 1[b] June 2020		Survey 2 July 2020		Survey 3 October 2020		Survey 4 January 2021		Survey 5 April 2021		Survey 6 September 2021		Survey 7 April 2022	
	n	% 95% CI	n	% 95% CI	n	% 95% CI	n	% 95% CI	n	% 95% CI	n	% 95% CI	n	% 95% CI
**Overall**	1183	11.4 (9.2-13.6)	1470	14.8 (12.1-17.6)	1129	18.6 (15.2-22.0)	1194	24.9 (21.0-28.7)	1187	38.1 (34.3-41.9)	1035	52.7 (48.7-56.8)	936	77.7 (74.4-81.0)
**Study strata (mean income)**	p<0.0001		p=0.2072		p=0.0083		p=0.0004		p=0.0022		p=0.0001		p<0.0001	
High	676	6.5 (4.4-8.5)	851	13.0 (9.2-16.8)	544	13.9 (9.6-18.2)	580	17.6 (12.7-22.6)	610	32.0 (26.7-37.4)	415	44.9 (39.2-50.5)	435	70.7 (65.1-76.2)
Low	507	16.0 (12.2-19.8)	619	16.5 (12.6-20.4)	585	23.0 (18.0-28.0)	614	31.9 (26.1-37.6)	577	44.0 (38.8-49.2)	620	60.4 (55.3-65.4)	501	84.2 (80.8-87.6)
**Sex**	p=0.8192		p=0.7207		p=0.6261		p=0.8207		p=0.3694		p=0.6701		p=0.8619	
Male	521	11.6 (8.7-14.5)	607	14.5 (10.9-18.0)	460	18.0 (13.2-22.8)	483	24.6 (19.4-29.7)	481	36.7 (31.7-41.7)	368	53.7 (46.9-60.5)	303	78.0 (73.3-82.6)
Female	662	11.2 (8.5-13.9)	863	15.1 (12.0-18.3)	669	19.2 (15.7-22.6)	711	25.1 (21.2-29.1)	706	39.4 (34.7-44.0)	667	51.9 (47.0-56.8)	633	77.5 (73.5-81.5)
**Age group (years)**	p=0.3585		p=0.0850		p=0.0237		p<0.0001		p=0.0001		p=0.0087		p<0.0001	
18 - 39	496	10.0 (7.1-12.8)	613	15.0 (11.7-18.4)	459	21.8 (17.2-26.4)	497	29.4 (24.4-34.3)	502	41.6 (36.3-47.0)	414	58.8 (53.3-64.3)	325	85.8 (81.6-90.0)
40 - 59	435	13.4 (9.7-17.1)	494	16.8 (12.3-21.2)	397	18.2 (13.2-23.3)	425	25.6 (20.6-30.6)	462	40.7 (35.6-45.8)	357	48.1 (41.7-54.4)	360	75.3 (69.7-80.9)
≥ 60	252	11.1 (6.0-16.2)	363	10.9 (7.4-14.5)	273	12.3 (7.5-17.2)	272	14.1 (9.3-19.0)	223	26.3 (20.5-32.1)	264	47.9 (40.7-55.1)	251	65.4 (58.1-72.6)
**Self-reported race/skin color**	p=0.0055		p=0.0543		p=0.0012		p<0.0001		p<0.0001		p=0.0011		p=0.0051	
White	614	7.9 (5.6-10.4)	733	12.9 (9.6-16.2)	556	14.4 (10.6-18.2)	609	18.0 (13.9-22.0)	537	30.9 (25.9-35.9)	406	45.3 (39.5-51.1)	435	72.1 (67.1-77.0)
Black or mixed (*Pardo*)	510	15.3 (11.9-18.7)	671	17.0 (13.2-20.7)	530	23.4 (19.0-27.8)	549	33.3 (27.9-38.7)	584	45.5 (40.8-50.2)	599	58.6 (53.9-63.3)	470	82.8 (79.0-86.5)
Asian or Indigenous	48	9.7 (0.0-20.6)	49	8.5 (1.6-15.4)	33	11.8 (0.6-23.0)	30	9.3 (0.0-19.9)	58	28.7 (15.9-41.5)	25	45.5 (21.6-69.3)	24	80.4 (60.5-100)
**Schooling (years)**	p=0.0004		p=0.0007		p=0.0001		p<0.0001		p<0.0001		p=0.0231		p=0.0469	
≥ 11	386	17.2 (12.2-22.2)	518	17.8 (13.9-21.7)	429	24.5 (18.7-30.3)	442	29.7 (23.6-35.9)	415	41.2 (35.4-47.0)	417	55.1 (49.9-60.3)	370	80.6 (76.5-84.7)
12 - 15	464	11.0 (8.4-13.5)	586	16.0 (12.2-19.8)	426	19.0 (14.6-23.4)	474	27.2 (22.7-31.7)	483	46.1 (40.7-51.6)	416	56.4 (51.1-61.7)	381	78.7 (73.8-83.6)
≥ 16	333	5.1 (1.8-8.4)	366	8.9 (5.9-11.9)	274	9.7 (6.0-13.4)	278	14.3 (9.7-18.9)	289	21.5 (15.6-27.4)	202	42.8 (33.0-52.6)	185	70.4 (62.5-78.3)

[a]In all surveys, seroprevalence estimates were weighted by sampling design with adjustments for non-response.

[b]Survey 1, the specimens were tested for IgM and IgG with the Maglumi SARS-CoV-2 test.
In Surveys 2 to Survey 7, the specimens were tested for anti-nucleocapsid total antibodies with the Roche Elecsys test.

Table 2 shows the growth model parameters for prevalence stratified by sex, age group and self-reported race/skin color, and Fig 3 presents the observed and modelled prevalence data for each of these variables. The overall trend of the point prevalence series (Fig 3a) identified two periods with significantly different growths: a monthly increase of approximately 2% (β1) in prevalence from June 2020 (Survey 1) to January 2021 (Survey 4) and a monthly increase of approximately 3.5% (β2) in prevalence from January 2021 (Survey 4) to April 2022 (Survey 7). For both sexes (Fig 3b), there is a joinpoint in January 2021 (Survey 4), and the overlap of the prevalence curves in the whole study period illustrates the lack of difference between the two groups. The trend analysis of the prevalence by age group (Fig 3c) indicated one joinpoint in January 2021 (Survey 4) for the 18-39 and 60 or older groups. After this point, both groups presented similar monthly increases (β2 = 3.5% and 3.7%, respectively), although the prevalence magnitudes differed. For the White self-reported race group, the set identified one joinpoint in January 2021 (Survey 4) and a significant increase of 3.5% between January 2021 and April 2022, whereas the Black or mixed self-reported race group showed a significant monthly increase of 3.1% over the whole study period (Fig 3d).

Table 2. Growth models and parameter estimates of point prevalence of SARS-CoV-2 antibody encompassing seven population-based surveys. The SoroEpi MSP Study, Municipality of São Paulo, SP, Brazil, June 2020 to April 2022.

	Period[a]	Parameter	Estimate	Standard Error	Test Statistic (t)	Prob > |t|
**Overall**						
	Jun 2020 - Jan 2021	α1	11.785	0.838	14.065	0.005
		β1	1.987*	0.445	4.463	0.047
	Jan 2021 - Apr 2022	α2	1.404	3.130	0.448	0.698
		β2	3.470*	0.183	18.958	0.003
**Study strata (mean income)**						
High	Jun 2020 - Apr 2022	α1	6.093	1.686	3.614	0.015
		β1	2.702*	0.221	12.239	0.000
Low	Jun 2020 - Oct 2020	α1	15.475	0.614	25.217	0.002
		β1	1.792	0.877	2.043	0.178
	Oct 2020 – Apr 2022	α2	8.947	1.050	8.518	0.014
		β2	3.424*	0.062	55.399	0.000
**Sex**						
Male	Jun 2020 - Jan 2021	α1	11.849	0.519	22.849	0.002
		β1	1.883*	0.294	6.415	0.023
	Jan 2021- Apr 2022	α2	0.210	1.999	0.105	0.926
		β2	3.546*	0.117	30.191	0.001
Female	Jun 2020 - Jan 2021	α1	11.815	1.229	9.611	0.011
		β1	2.056	0.586	3.508	0.073
	Jan 2021 - Apr 2022	α2	2.337	4.713	0.496	0.669
		β2	3.410*	0.273	12.469	0.006
**Age group (years)**						
18-39	Jun 2020 - Jan 2021	α1	10.895	0.931	11.704	0.007
		β1	2.726*	0.522	5.219	0.035
	Jan 2021 - Apr 2022	α2	4.006	3.718	1.078	0.394
		β2	3.710*	0.212	17.502	0.003
40-59	Jun 2020 - Apr 2022	α1	11.563	1.998	5.789	0.002
		β1	2.725*	0.208	13.087	0.000
≥ 60	Jun 2020 - Jan 2021	α1	10.458	1.593	6.563	0.022
		β1	0.652	0.733	0.890	0.467
	Jan 2021 - Apr 2022	α2	-9.737	5.423	-1.795	0.214
		β2	3.537*	0.348	10.170	0.010

[a]The JoinPoint analysis identified the number of turning points and period (trend segment) for each variable.

*Slope significantly different from zero at the alpha=0.05 level.

Fig 3. Joinpoint trends for SARS-CoV-2 seropositivity in the Municipality of São Paulo according to surveys from June 2020 to April 2022. a) overall, b) sex, c) age group, and d) self-reported race/skin color.

Table 3 shows the estimated population who had had SARS-CoV-2 infection in the pre-vaccination period (Surveys 1 to 4) in the municipality of São Paulo, the number of cumulative reported COVID-19 cases and the infection-to-case ratio. The estimated number of persons who were infected ranged from 1.1 million in June 2020 (Survey 1) to approximately 2.3 million in January 2021 (Survey 4). The underreporting multiplier varied from 8.9 in Survey 1 to 4.3 in Survey 4.

Table 3. Estimated number of adults infected with SARS-CoV-2 and the ratio of estimated infections by reported COVID-19 cases in the pre-vaccination period, The SoroEpi MSP Study, Municipality of São Paulo, SP, Brazil, June 2020 to January 2021.

Survey (specimen collection period)	Population[a]	Sample size n	SARS-CoV-2 seroprevalence % (95% CI)	Estimated number of adults with SARS-CoV-2 infection n (95% CI)	Cumulative reported COVID-19 cases (date)[b]	Ratio (infections:cases)
Survey 1 (15-24 June 2020)	9,164,926	1,183	11.4 (9.2-13.6)	1,044,802 (843,173-1,246,430)	117,768 (20 June)	8.9 (7.2-10.6)
Survey 2 (20-29 July 2020)	9,164,926	1,470	14.8 (12.1-17.6)	1,356,409 (1,108,956-1,613,027)	203,308 (24 July)	6.7 (5.5-7.9)
Survey 3 (1-10 October 2020)	9,164,926	1,129	18.6 (15.2-22.0)	1,704,676 (1,393,069-2,016,284)	337,206 (5 October)	5.1 (4.1-6.0)
Survey 4 (14-23 January 2021)	9,216,158	1,194	24.9 (21.0-28.7)	2,294,823 (1,935,393-2,645,037)	528,809 (18 January)	4.3 (3.7-5.0)

[a]Population aged 18 years and older projected by Fundação SEADE for the municipality of São Paulo.

[b]Source: Secretaria Municipal de Saúde, São Paulo.

Discussion

The present study presents new data on the seroprevalence trends of SARS-CoV-2 antibodies in the general adult population of the municipality of São Paulo, Brazil, based on seven cross-sectional surveys over an almost 2-year period. Our data showed that SARS-CoV-2 seropositivity increased over time, ranging from 11.4% (95%CI: 9.2-13.6) in June 2020 to 77.7% (95%CI: 74.4-81.0) in April 2022. We observed a significantly higher increase in the seroprevalence trend from January 2021 to April 2022 compared to 2020, most probably driven by the implementation of the COVID-19 vaccination campaigns.

Our results revealed that approximately 80% of the adult population in the municipality of São Paulo was susceptible to SARS-CoV-2 infection before vaccine availability in January 2021. A relatively small variation, from 11.4% (95%CI: 9.2-13.6) in June 2020 to 18.6% (95%CI: 15.2-22.0) in October 2020, measured natural immunity using an anti-nucleocapsid antibody test. In the pre-COVID-19 vaccine era, serosurveys using rapid tests (lateral flow Wondfo Biotech) in the city of São Paulo showed an even lower seroprevalence of SARS-CoV-2 (13.6%) by September 2020 [14]. Buss et al reported 44% of prevalence of IgG antibodies to SARS-CoV-2 nucleocapsid (N) protein (Abbot) among blood donors in the city of Manaus (Brazilian Amazon) by June 2020 [26]. The prevalence rate was higher in the Amazon than in São Paulo (13.6%, 95% CI: 12.0-18.1) in the same period, showing distinct patterns of the epidemic within the country [26]. The prevalence reported among blood donors is similar to the 11.4% (95% CI: 9.2-13.6) found in the general population in June 2020 (Survey-1). A serial cross-sectional SARS-CoV-2 antibody (Abbot assay) among blood

donors showed a range of adjusted seroprevalence from 19.0% (95% CI 15.9-22.4) in December 2020 to 22.3% (95%CI19.0-25.9) in February 2021 in the municipality of São Paulo [27]. These findings are in line with our population-based level of 24.9% (95% CI 21.0-28.7) of weighted seroprevalence of anti-SARS-CoV-2 in January 2021 (Survey-4), which is within the time period analysed by Prete et al [27]. However, comparison may be hampered by differences in the assays performed and the study populations. A systematic review and pooled analysis estimated that around 10% of the Brazilian population had been infected by March 2021 [28]. Globally, there was a large variation in prevalence among the geographic regions over time, and the authors called attention to the need for continuous monitoring of the population’s immunity levels [12,28,29].

The high level of seropositivity in the adult population was only reached by the date of our last survey (April 2022). This population-level immunity reflected the past virus infection and/or vaccination, mostly with CoronaVac, eliciting anti-nucleocapsid antibodies. The high level of hybrid immunity was mainly achieved by extensive vaccination coverage. We acknowledge that the reported positivity of SARS-CoV-2 antibodies at the population level did not include the anti-Spike antibodies elicited by other vaccine types [30]. In this sense, we may assume that the overall seroprevalence in our study could be underestimated. A seroprevalence of more than 95%, using SARS-CoV-2 anti-Spike protein assay, was reported among blood donors in the city of São Paulo and six other Brazilian capitals in November 2021 [31]. Our results were similar to those observed in serial surveys among blood donors in the US, where the seroprevalence induced by hybrid immunity reached more than 80% [32]. In our study, adults living in

districts classified as low-income stratum, and those who self-declared Black or mixed (*Pardo)*, were the most infected over the entire study period, in line with other serosurveys conducted in Brazil [14,33] and elsewhere [28]. In our setting, during 2020, higher COVID-19 mortality was observed among the lower-income stratum and Black populations, showing marked social inequalities [34,35].

In our models, the upward trend of the overall seroprevalence and the seroprevalence estimates stratified by sex, age group and self-reported race, indicated that January 2021 was the inflection point in the study period. The overlapping of the growth curves for males and females suggested similarities in the frequency of SARS-CoV-2 viral infection as the result of natural immunity in the year 2020 and/or vaccine-induced immunity afterwards. People over 60 years of age showed low seroprevalence, around 10%, with no significant increase in the year 2020, in contrast to significant increases among the younger age groups. This likely reflected the higher compliance to lockdown or social distance measures among the older population reported in other Brazilian regions [36]. Considering the fitted models for those aged 60 years or older, there was around a threefold increase in the slope estimated after January 2021. This high slope in prevalence growth occurred concurrently with the prioritization of COVID-19 vaccination among the older population. Therefore, vaccination seems to be the likely explanation for the rapid increase in hybrid immunity in older individuals. Of note, approximately half of the older population received the CoronaVac vaccine, the first vaccine product available in Brazil.

We found at least 8.9 (June 2020) and 4.3 (January 2021) times higher numbers of SARS-CoV-2 infections than the number of COVID-19 cases reported by the official surveillance system in 2020. These figures aligned with

the 6.9 overall estimated ratio of the SARS-CoV-2 infection by the confirmed COVID-19 reported cases in the region of the Americas, based mainly on US serosurveys [37]. Several possible explanations could be offered for the variation of the infections:reported cases ratio in our setting. Asymptomatic or mild symptomatic cases were seldom reported; laboratory tests for disease confirmation were restricted to severe cases in the early phases of the epidemic; and there were changes in the criteria for reporting COVID-19 cases, with cases being confirmed based on symptoms and imaging tests [38].

Population-based seroprevalence surveys of SARS-CoV-2 can provide estimates of the magnitude of SARS-CoV-2 infections irrespective of case-based surveillance [39]. In the UK, the Office for National Statistics has been performing repeated antibody and RT-PCR swab surveys since 2020, in addition to gathering case-surveillance data [40]. Also, in the US, a large national probabilistic repeated household survey has been in place to estimate the cumulative incidence in the stages of the COVID-19 pandemic [41].

SARS-CoV-2 was difficult to control and still imposes challenging uncertainties [42]. Sequential population-based studies with probabilistic sampling and highly accurate laboratory assays are central to monitoring the infection's complex and changing population dynamics.

To our knowledge, this was the only seven-round study of the point prevalence of SARS-CoV-2 antibodies in a representative sample of the general adult population of the municipality of São Paulo. It included the pre- and post-vaccination periods throughout the two years of the pandemic. A key strength of our study was the use of a robust methodology including the probability sampling

method to select the survey participants, together with statistical weighting and adjustments for demographics and non-response. The use of a highly sensitive, specific and fully automated assay to detect anti-nucleocapsid SARS-CoV-2 immunoglobulins was another strength. Moreover, the same rigorous methodology, data analysis strategies, and testing procedures were repeated in all surveys, despite the adverse conditions such as periods of lockdown and social distancing.

Although our study has significant strengths, several limitations must be acknowledged. The first limitation was that we did not include people under 18 years old due to logistic, time, and cost restrictions. Second, like most population-based household serosurveys [12], our study had a low household response rate. Third, we used an anti-SARS-CoV-2-nucleocapsid test that did not distinguish between antibodies induced by natural infection and those induced by vaccines. Fourth, seroprevalence results in Survey 1 were derived from the available laboratory test at the time, which was not the same as the test used in Surveys 2-7. Since there was only a one-month interval between Survey 1 and Survey 2, and both yielded similar estimates, we judged that it did not introduce measurement bias. Fifth, we did not consider the antibody waning over the months following infection, which could have led to underestimated results. However, we used Roche Elecsys immunoassay, which is considered a high sensitivity, high specificity assay and less prone to antibody waning [43]. Our study design which consisted of seven independent population-based surveys did not allow us to measure antibody waning directly as in the cohort studies. Last, it is important to recognize that the joinpoint trend analysis is usually

performed in longer series of point estimates composed of higher numbers of observations.

Conclusion

Our results documented the evolution of the SARS-CoV-2 infection patterns and vaccine response throughout the two years of the pandemic in the municipality of São Paulo. It enabled us to learn about the epidemiological features of the infection, evidencing the complexity of the population-based seropositivity profile. Our study reinforced the importance of conducting repeated serological surveillance to monitor and study how new infectious pathogen transmission dynamics evolve in susceptible populations. Such data may add to the understanding of the complex and changing population dynamics of the SARS-CoV-2 infection, including the impact of vaccination strategies and the modelling of future epidemiological scenarios.

Acknowledgments

We thank all the study participants whose cooperation made the study possible.

References

1. Castro MC, Kim S, Barberia L, Ribeiro AF, Gurzenda S, Ribeiro KB, et al. Spatiotemporal pattern of COVID-19 spread in Brazil. Science. 2021;372: 821–826. doi:10.1126/science.abh1558
2. WHO Health Emergency Dashboard. WHO Coronavirus (COVID-19) Dashboard. [cited 8 Nov 2022]. Available: https://covid19.who.int
3. WHO Health Emergency Dashboard. Brazil: WHO Coronavirus Disease (COVID-19) Dashboard With Vaccination Data. [cited 8 Nov 2022]. Available: https://covid19.who.int/region/amro/country/br
4. Worldometer. Brazil Population (2022). [cited 8 Nov 2022]. Available: https://www.worldometers.info/world-population/brazil-population/
5. Góes C, Karpowicz I. Inequality in Brazil: A Regional Perspective. 2017;2017: 34. doi:https://doi.org/10.5089/9781484324776.001
6. IBGE. Instituto Brasileiro de Geografia e Estatística. Projeções da População IBGE. [cited 7 Nov 2022]. Available: https://www.ibge.gov.br/estatisticas/sociais/populacao/9109-projecao-da-populacao.html?=&t=resultados
7. Candido DDS, Watts A, Abade L, Kraemer MUG, Pybus OG, Croda J, et al. Routes for COVID-19 importation in Brazil. J Travel Med. 2020;27: taaa042. doi:10.1093/jtm/taaa042
8. Candido DS, Claro IM, de Jesus JG, Souza WM, Moreira FRR, Dellicour S, et al. Evolution and epidemic spread of SARS-CoV-2 in Brazil. Science. 2020;369: 1255–1260. doi:10.1126/science.abd2161
9. Secretaria Municipal da Saúde. Prefeitura da Cidade de São Paulo. Boletim Semanal Covid-19 no município de São Paulo de 14 de setembro de 2022. Prefeitura da Cidade de São Paulo; 14 Sep 2022 [cited 21 Dec 2022]. Available: https://www.prefeitura.sp.gov.br/cidade/secretarias/saude/vigilancia_em_saude/doencas_e_agravos/coronavirus/index.php?p=335024
10. Bryant JE, Azman AS, Ferrari MJ, Arnold BF, Boni MF, Boum Y, et al. Serology for SARS-CoV-2: Apprehensions, opportunities, and the path forward. Sci Immunol. 2020;5: eabc6347. doi:10.1126/sciimmunol.abc6347
11. Murhekar MV, Bhatnagar T, Selvaraju S, Saravanakumar V, Thangaraj

JWV, Shah N, et al. SARS-CoV-2 antibody seroprevalence in India, August–September, 2020: findings from the second nationwide household serosurvey. Lancet Glob Health. 2021;9: e257–e266. doi:10.1016/S2214-109X(20)30544-1

12. Bergeri I, Whelan MG, Ware H, Subissi L, Nardone A, Lewis HC, et al. Global SARS-CoV-2 seroprevalence from January 2020 to April 2022: A systematic review and meta-analysis of standardized population-based studies. PLOS Med. 2022;19: e1004107. doi:10.1371/journal.pmed.1004107
13. Hallal PC, Hartwig FP, Horta BL, Silveira MF, Struchiner CJ, Vidaletti LP, et al. SARS-CoV-2 antibody prevalence in Brazil: results from two successive nationwide serological household surveys. Lancet Glob Health. 2020;8: e1390–e1398. doi:10.1016/S2214-109X(20)30387-9
14. Albuquerque JOM de, Kamioka GA, Madalosso G, Costa SA, Ferreira PB, Pino FA, et al. Prevalence evolution of SARS-CoV-2 infection in the city of São Paulo, 2020–2021. Rev Saúde Pública. 2021;55: 62–62. doi:10.11606/s1518-8787.2021055003970
15. Tess BH, Granato CFH, Alves MCGP, Pintão MCT, Nunes MC, Rizzatti EG, et al. Assessment of initial SARS-CoV-2 seroprevalence in the most affected districts in the municipality of São Paulo, Brazil. Braz J Infect Dis. 2021;25. doi:10.1016/j.bjid.2021.101604
16. FIOCRUZ. FIOCRUZ: Genomic Network. In: Genomahcov - Fiocruz [Internet]. [cited 9 Feb 2023]. Available: https://www.genomahcov.fiocruz.br/dashboard-pt/
17. WHO. Population-based age-stratified seroepidemiological investigation protocol for coronavirus 2019 (COVID-19) infection. World Health Organ. 2020; 20.
18. SEADE. Fundação Sistema Estadual de Análise de Dados. Sistema SEADE de Projeções Populacionais. [cited 7 Nov 2022]. Available: http://produtos.seade.gov.br/produtos/projpop/
19. IBGE. Instituto Brasileiro de Geografia e Estatística. Desigualdades sociais por cor ou raça no Brasil. [cited 20 Dec 2022]. Available: https://biblioteca.ibge.gov.br/index.php/biblioteca-catalogo?view=detalhes&id=2101972

20. Muench P, Jochum S, Wenderoth V, Ofenloch-Haehnle B, Hombach M, Strobl M, et al. Development and validation of the Elecsys Anti-SARS-CoV-2 immunoassay as a highly specific tool for determining past exposure to SARS-CoV-2. J Clin Microbiol. 2020;58.
21. Padoan A, Cosma C, Sciacovelli L, Faggian D, Plebani M. Analytical performances of a chemiluminescence immunoassay for SARS-CoV-2 IgM/IgG and antibody kinetics. Clin Chem Lab Med. 2020;58: 1081–1088. doi:10.1515/cclm-2020-0443
22. Peluso MJ, Takahashi S, Hakim J, Kelly JD, Torres L, Iyer NS, et al. SARS-CoV-2 antibody magnitude and detectability are driven by disease severity, timing, and assay. Sci Adv. 2021;7: eabh3409. doi:10.1126/sciadv.abh3409
23. WHO. Interim statement on hybrid immunity and increasing population seroprevalence rates. 1 Jun 2022 [cited 21 Dec 2022]. Available: https://www.who.int/news/item/01-06-2022-interim-statement-on-hybrid-immunity-and-increasing-population-seroprevalence-rates
24. Statistical Methodology and Applications Branch, Surveillance Research Program, National Cancer Institute. Joinpoint Regression Program. 2022. Available: https://surveillance.cancer.gov/joinpoint/
25. Kim HJ, Fay MP, Feuer EJ, Midthune DN. Permutation tests for joinpoint regression with applications to cancer rates. Stat Med. 2000;19: 335–351. doi:10.1002/(sici)1097-0258(20000215)19:3<335::aid-sim336>3.0.co;2-z
26. Buss LF, Prete CA, Abrahim CMM, Mendrone A, Salomon T, de Almeida-Neto C, et al. Three-quarters attack rate of SARS-CoV-2 in the Brazilian Amazon during a largely unmitigated epidemic. Science. 2021;371: 288–292. doi:10.1126/science.abe9728
27. Prete CA, Buss LF, Whittaker C, Salomon T, Oikawa MK, Pereira RHM, et al. SARS-CoV-2 antibody dynamics in blood donors and COVID-19 epidemiology in eight Brazilian state capitals: A serial cross-sectional study. eLife. 2022;11: e78233. doi:10.7554/eLife.78233
28. Metzger C, Leroy T, Bochnakian A, Jeulin H, Gegout-Petit A, Legrand K, et al. Seroprevalence and SARS-CoV-2 invasion in general populations: A scoping review over the first year of the pandemic. PLOS ONE. 2023;18: e0269104. doi:10.1371/journal.pone.0269104

29. Rostami A, Sepidarkish M, Fazlzadeh A, Mokdad AH, Sattarnezhad A, Esfandyari S, et al. Update on SARS-CoV-2 seroprevalence: regional and worldwide. Clin Microbiol Infect. 2021;27: 1762–1771. doi:10.1016/j.cmi.2021.09.019
30. Duarte N, Yanes-Lane M, Arora RK, Bobrovitz N, Liu M, Bego MG, et al. Adapting Serosurveys for the SARS-CoV-2 Vaccine Era. Open Forum Infect Dis. 2022;9: ofab632. doi:10.1093/ofid/ofab632
31. Buss L, Prete CA, Whittaker C, Salomon T, Oikawa MK, Pereira RHM, et al. Predicting SARS-CoV-2 Variant Spread in a Completely Seropositive Population Using Semi-Quantitative Antibody Measurements in Blood Donors. Vaccines. 2022;10: 1437. doi:10.3390/vaccines10091437
32. Jones JM, Stone M, Sulaeman H, Fink RV, Dave H, Levy ME, et al. Estimated US Infection- and Vaccine-Induced SARS-CoV-2 Seroprevalence Based on Blood Donations, July 2020-May 2021. JAMA. 2021;326: 1400–1409. doi:10.1001/jama.2021.15161
33. Barros AJD, Victora CG, Menezes AMB, Horta BL, Barros FC, Hartwig FP, et al. Population-level seropositivity trend for SARS-Cov-2 in Rio Grande do Sul, Brazil. Rev Saúde Pública. 2021;55. doi:10.11606/s1518-8787.2021055004075
34. Ribeiro KB, Ribeiro AF, Veras MA de SM, de Castro MC. Social inequalities and COVID-19 mortality in the city of São Paulo, Brazil. Int J Epidemiol. 2021;50: 732–742. doi:10.1093/ije/dyab022
35. Li SL, Pereira RHM, Jr CAP, Zarebski AE, Emanuel L, Alves PJH, et al. Higher risk of death from COVID-19 in low-income and non-White populations of São Paulo, Brazil. BMJ Glob Health. 2021;6: e004959. doi:10.1136/bmjgh-2021-004959
36. Carvalho MS, Bastos LS, Fuller T, Cruz OG, Damasceno L, Calvet G, et al. Incidence of SARS-CoV-2 over four epidemic waves in a low-resource community in Rio de Janeiro, Brazil: A prospective cohort study. Lancet Reg Health – Am. 2022;12. doi:10.1016/j.lana.2022.100283
37. Chen X, Chen Z, Azman AS, Deng X, Sun R, Zhao Z, et al. Serological evidence of human infection with SARS-CoV-2: a systematic review and meta-analysis. Lancet Glob Health. 2021;9: e598–e609. doi:10.1016/S2214-109X(21)00026-7

38. Barberia LG, Cantarelli LGR, Oliveira MLC de F, Moreira N de P, Rosa ISC. The effect of state-level social distancing policy stringency on mobility in the states of Brazil. Rev Adm Pública. 2021;55: 27–49. doi:10.1590/0034-761220200549
39. Shook-Sa BE, Boyce RM, Aiello AE. Estimation Without Representation: Early Severe Acute Respiratory Syndrome Coronavirus 2 Seroprevalence Studies and the Path Forward. J Infect Dis. 2020;222: 1086–1089. doi:10.1093/infdis/jiaa429
40. Office for National Statistics (ONS). Coronavirus (COVID-19) Infection Survey, antibody data, UK: 5 October 2022. ONS website; 2022 Oct. Available: https://www.ons.gov.uk/peoplepopulationandcommunity/healthandsocialcare/conditionsanddiseases/bulletins/coronaviruscovid19infectionsurveyantibodyandvaccinationdatafortheuk/5october2022
41. National Institutes of Health. NIH begins study to quantify undetected cases of coronavirus infection. In: National Institutes of Health (NIH) [Internet]. 10 Apr 2020 [cited 7 Nov 2022]. Available: https://www.nih.gov/news-events/news-releases/nih-begins-study-quantify-undetected-cases-coronavirus-infection
42. Anderson RM, Vegvari C, Hollingsworth TD, Pi L, Maddren R, Ng CW, et al. The SARS-CoV-2 pandemic: remaining uncertainties in our understanding of the epidemiology and transmission dynamics of the virus, and challenges to be overcome. Interface Focus. 11: 20210008. doi:10.1098/rsfs.2021.0008
43. Muecksch F, Wise H, Batchelor B, Squires M, Semple E, Richardson C, et al. Longitudinal Serological Analysis and Neutralizing Antibody Levels in Coronavirus Disease 2019 Convalescent Patients. J Infect Dis. 2021;223: 389–398. doi:10.1093/infdis/jiaa659

Supporting information

S1 Table. Frequency of selected characteristics of the sample population per survey. The SoroEpi MSP Study, Municipality of São Paulo, SP, Brazil, June 2020 to April 2022.

S2 Table. Frequency of adults (and 95% Confidence Interval) by type of anti-SARS-CoV-2 vaccine received and age group in Surveys 5, 6 and 7. The SoroEpi MSP Study, Municipality of São Paulo, SP, Brazil, June 2020 to April 2022.

Figure 1

Click here to access/download;Figure;Fig1.tiff

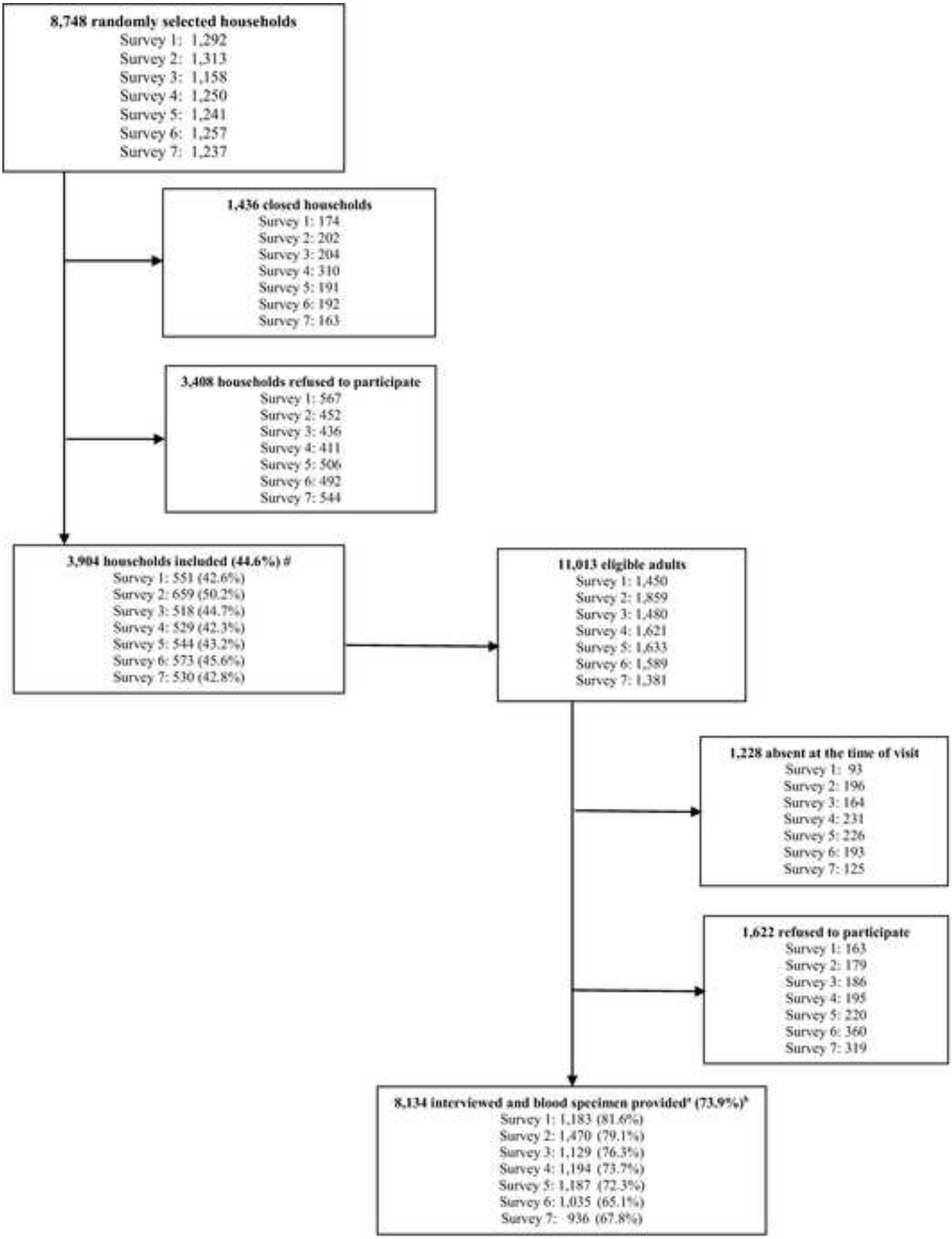

[a]19 individuals excluded due to insufficient blood specimen: Survey 1: 11; Survey 2: 14; Surveys 3, 4, 6, 7: 1
[b]Overall participation rate: 38.4% (95% CI: 37.7-39.1)

Figure 2

Click here to access/download;Figure;Fig2.tiff

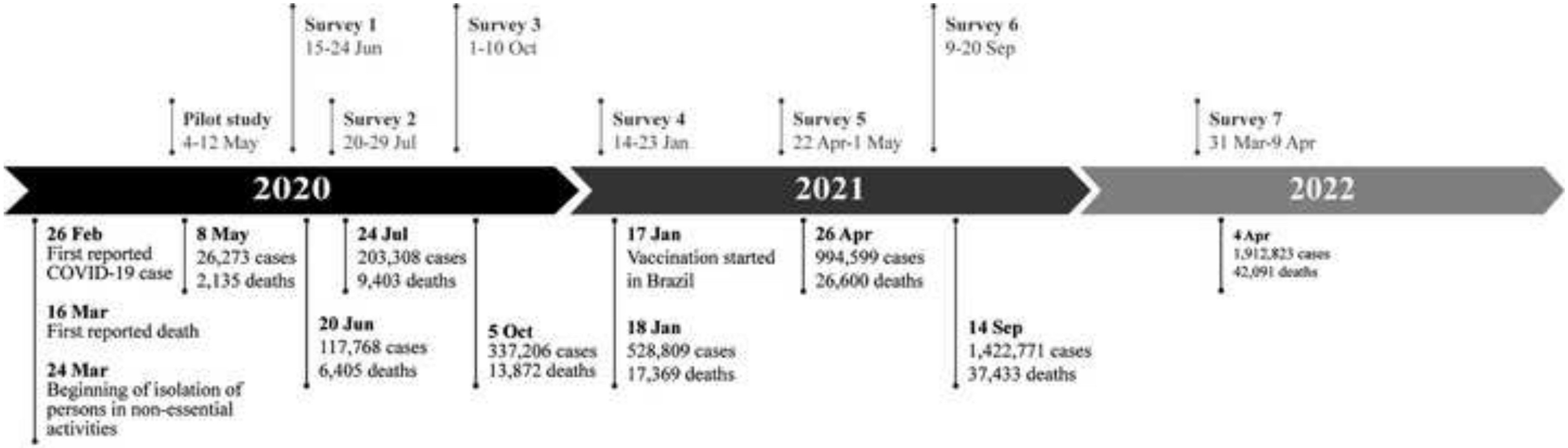

Reported cumulative COVID-19 cases and deaths available at: https://www.prefeitura.sp.gov.br/cidade/secretarias/saude/vigilancia_em_saude/index.php?p=295572.
Reported cases were laboratory confirmed until March 2021 and afterwards, cases were also reported by clinical epidemiological criteria.

Figure 3a

Click here to access/download;Figure;Fig3a.tiff

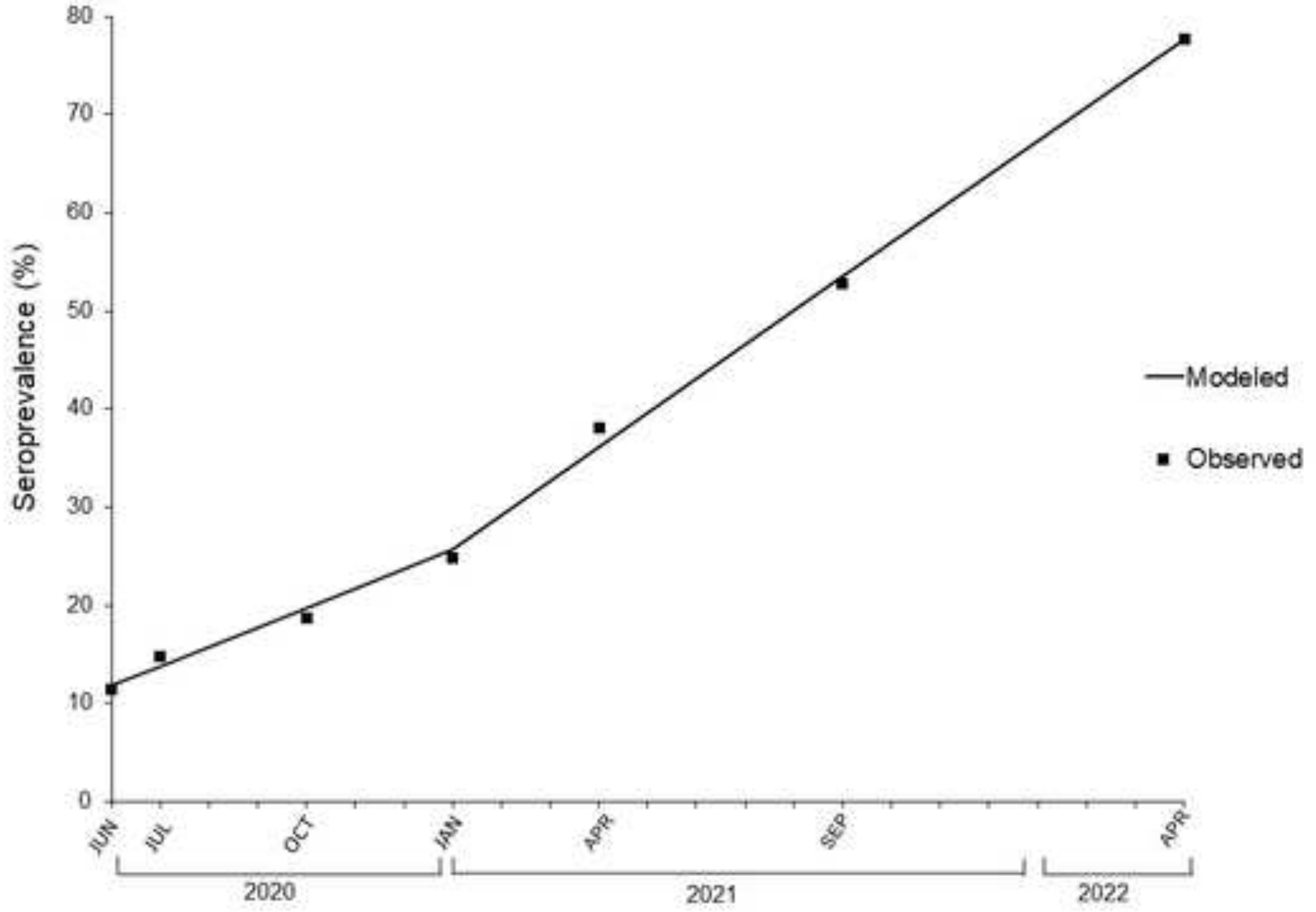

Figure 3b

Click here to access/download;Figure;Fig3b.tiff

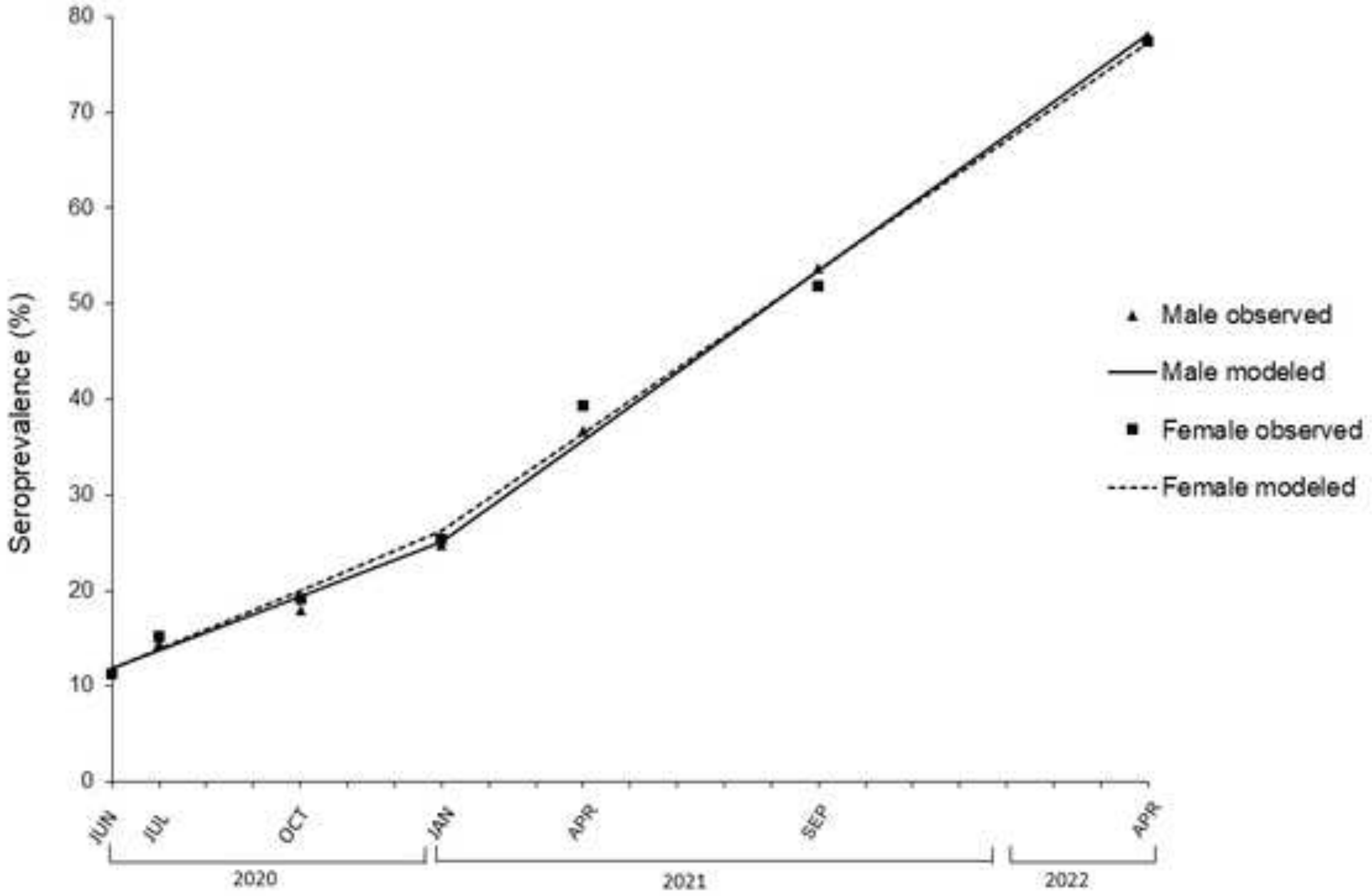

Figure 3c

Click here to access/download;Figure;Fig3c.tiff

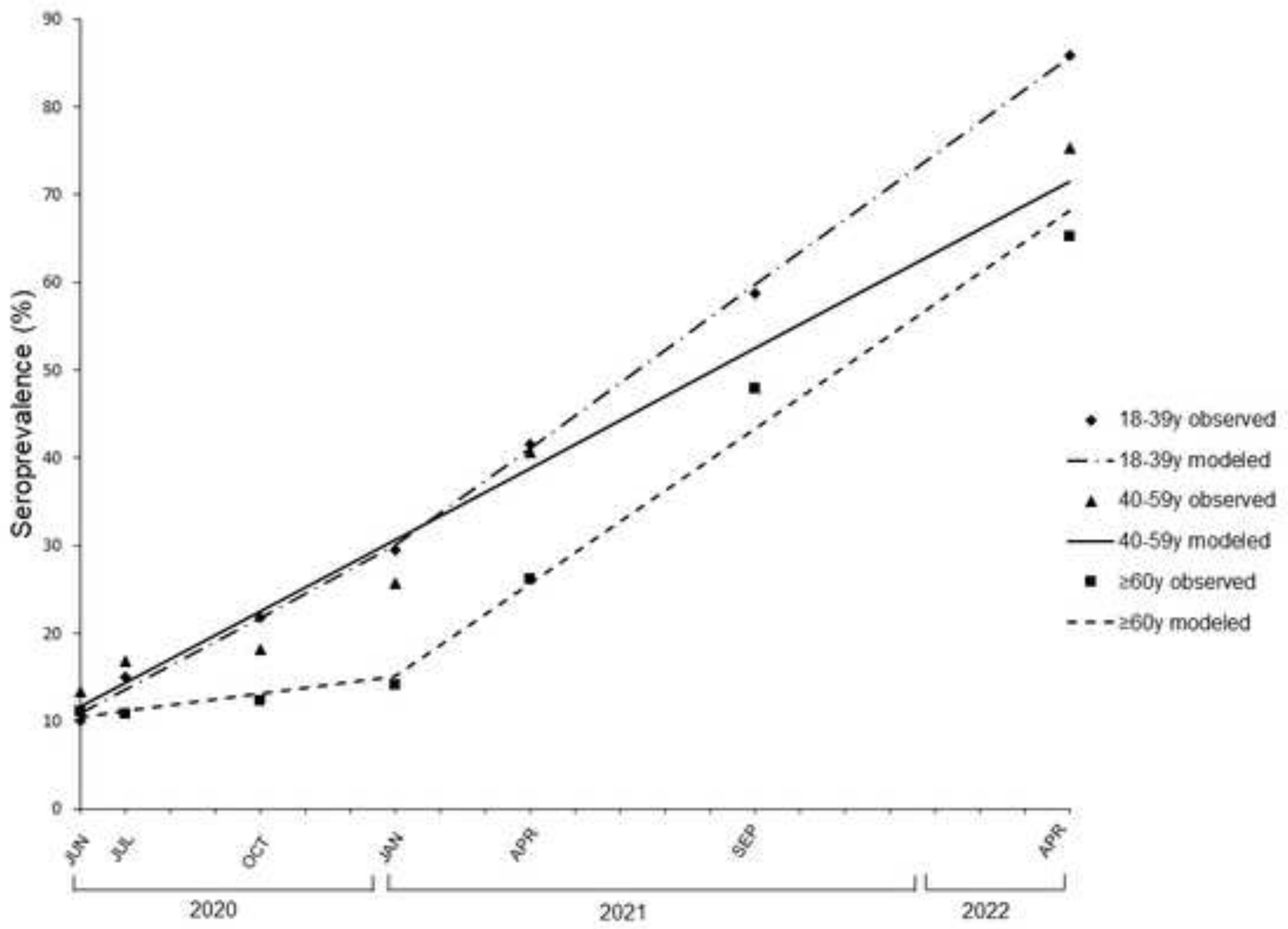

Figure 3d

Click here to access/download;Figure;Fig3d.tiff

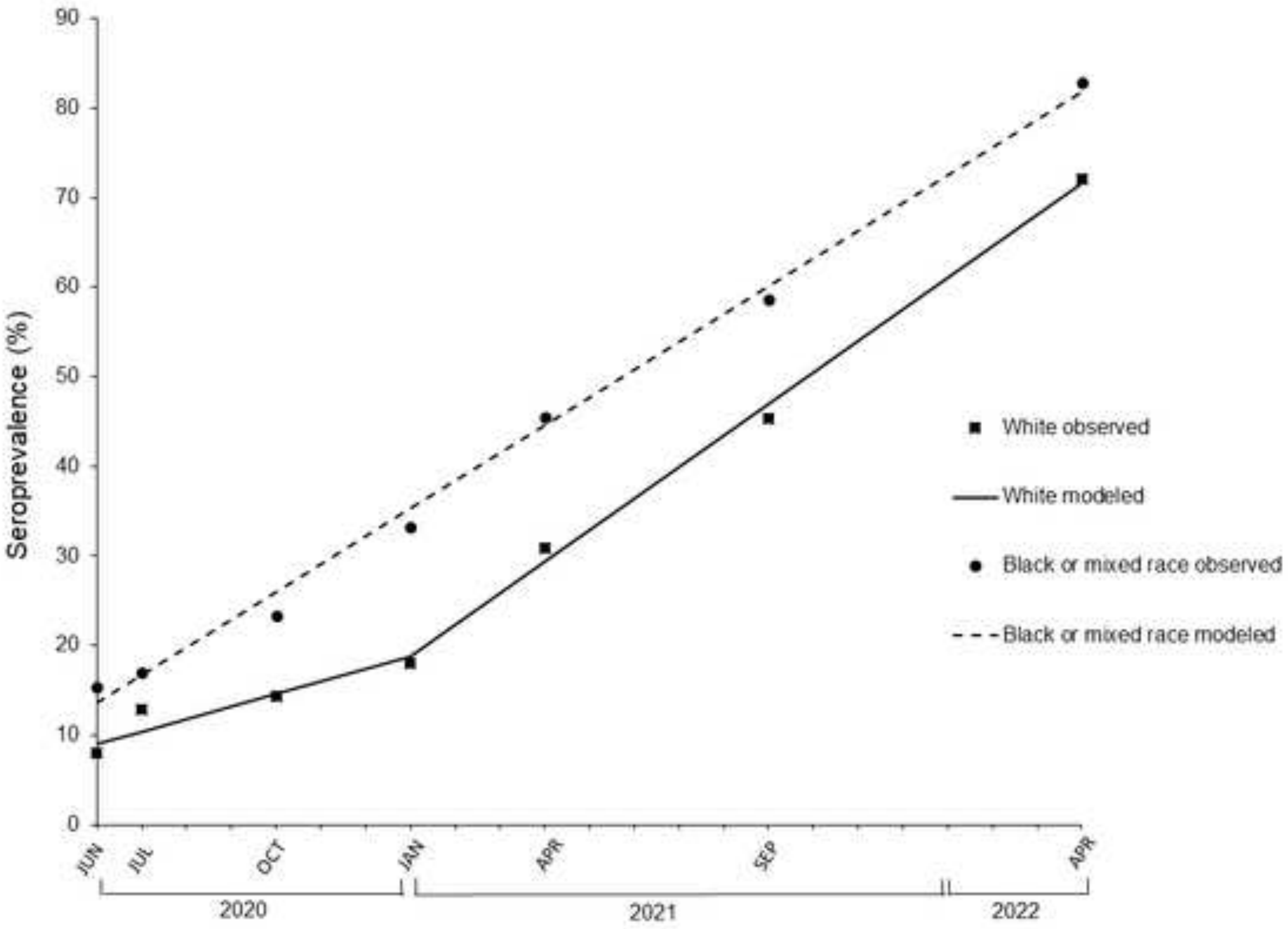

Supplementary table 1

Click here to access/download
Supporting Information
S1_Tab.docx

Supplementary table 2

Click here to access/download
Supporting Information
S2_Tab.docx

Seroprevalence trends of anti-SARS-CoV-2 antibodies in the adult population of the São Paulo Municipality, Brazil: Results from seven serosurveys from June 2020 to April 2022. The SoroEpi MSP Study

Formatted: Portuguese (Brazil)

Beatriz Helena Tess[1*¶^], Celina Maria Turchi Martelli[2¶], Maria Cecília Goi Porto Alves[3^], Fanny Cortes[4], Regina Tomie Ivata Bernal[5], Wayner Vieira de Souza[2], Expedito José de Albuquerque Luna[1], Laura da Cunha Rodrigues[6], Marcia Cavallari Nunes[7^], Fernando de Castro Reinach[8^], Celso Francisco Hernandes Granato[9^], Edgar Gil Rizzatti[9^], Maria Carolina Tostes Pintão[9^]

[1]Departamento de Medicina Preventiva, Faculdade de Medicina, Universidade de São Paulo, São Paulo, SP, Brasil

[2]Instituto Aggeu Magalhães, Fiocruz, Recife, PE, Brasil

[3]Instituto de Saúde, Secretaria de Saúde do Estado de São Paulo, São Paulo, SP, Brasil

[4]Instituto de Avaliação de Tecnologia em Saúde, Porto Alegre, RS, Brasil

[5]Programa de Pós-Graduação, Escola de Enfermagem, Universidade Federal de Minas Gerais, Belo Horizonte, MG, Brasil

[6]Department of Infectious Disease Epidemiology, London School of Hygiene & Tropical Medicine, London, UK

[7]Inteligência em Pesquisa e Consultoria (IPEC), São Paulo, SP, Brasil

[8]Departamento de Bioquímica, Instituto de Química, Universidade de São Paulo, São Paulo, SP, Brasil

[9]Divisão de Pesquisa e Desenvolvimento, Grupo Fleury, São Paulo, SP, Brasil

*Corresponding author

E-mail: beatriz.tess@usp.br (BHT)

[¶]These authors contributed equally to this work.

^Members of the SoroEpi MSP Study Research Group.

Formatted: Portuguese (Brazil)

Field Code Changed

Formatted: Underline, Font color: Blue, Portuguese (Brazil)

Formatted: Portuguese (Brazil)

Abstract

Formatted: Font: 18 pt

Background: Sequential population-based household serosurveys of SARS-CoV-2 cover~~ompris~~ing the COVID-19 pre- and post-vaccination periods are scarce in Brazil. This study aimed to investigate trends of seropositivity among adults in the municipality of São Paulo, Brazil.

Methods: We conducted seven cross-sectional surveys of adult population-representative samples between June 2020 and April 2022. The study design included probabilistic sampling, assessment of the presence of SARS-CoV-2 antibodies using~~with~~ the Roche Elecsys anti-nucleocapsid assay, and statistical adjustments for ~~the ~~population demographics and non-response. The weighted seroprevalences with 95% confidence intervals (CI) were estimated by sex, age group, race, schooling, and mean income study strata. Time trends in seropositivity were assessed using the Joinpoint model. We compared infection-induced seroprevalences with COVID-19 reported cases.

Results: The study sample comprised 8,134 adults~~ composed the study sample~~. The overall SARS-CoV-2 seroprevalence increased from 11.4% (95%CI: 9.2-13.6) in June 2020 to 24.9% (95%CI: 21.0-28.7) in January 2021; and from 38.1% (95%CI: 34.3-41.9) in April 2021 to 77.7% (95%CI: 74.4-81.0) in April 2022. The prevalence over time was higher in the subgroups ~~of ~~18–39-years of age ~~-old-age ~~and self-reported Black or mixed (*P~~p~~ardo*) race. Monthly prevalence rose steeply from January 2021 onwards, particularly among those aged 60 years or

older, who were prioritized for vaccination~~receiving vaccines~~. The infection-to-case ratios ranged from 8.9 in June 2020 to 4.3 in January 2021.

Conclusions: We observed that the overall seroprevalence rose significantly over time and with age and race subgroup~~s~~ variations. Increases in the 60 years or older age ~~-~~and the White adult~~s~~ groups after the beginning of the vaccination campaign were faster than in younger ages and Black or mixed (*P~~p~~ardo*) race groups. Our data may add to the understanding of the complex and changing population dynamics of the SARS-CoV-2 infection, including the impact of vaccination strategies and the modelling of future epidemiological scenarios.

Keywords

SARS-CoV-2, COVID-19, prevalence, serosurvey, adult, general population, Brazil

Introduction

Brazil was one of the countries most affected by the COVID-19 pandemic [1]. From February 2020 to 20 September 2022, approximately 34.6 million confirmed cases of COVID-19, with 685,000 ~~thousand~~ deaths, were reported [2]. During that period, Brazil contributed to 5.6% of the global COVID-19 cases and around 10% of the total reported deaths [3], even though it~~s~~ ~~population~~ accounts for only~~represents~~ 2.3% of the world population [4].

São Paulo is among the top ten metropolitan areas of the world. It is in the wealthiest Brazilian region and has a high level of socioeconomic inequality [5]. The city of São Paulo, with a population of approximately 11.9 million [6], was the site of SARS-CoV-2 entry~~ance~~ into the country, the initial epicenter of the pandemic, and an important route of viral spread [7,8]. As of 14 September 2022, the São Paulo Municipality reported 2,281,679 confirmed cases and 43,785 deaths [9].

Cross-sectional surveys are snapshots of infection history. They can complement ~~the~~ data based on PCR-confirmed cases by identifying ~~the~~ asymptomatic infections and the magnitude of transmission in different settings and populations [10]. Repeated serosurveys can monitor the spread of the virus in the general population and describe the geographical and subgroup changes over time. Knowledge of trends of SARS-CoV-2 infections can inform and help evaluate the impact of public health actions to~~ward~~ control~~ling~~ the pandemic~~s~~ and shed light on the dynamics of the interaction between the virus and the population~~s~~ [11].

A systematic review of global SARS-CoV-2 seroprevalence from January 2020 to May 2022 indicated that most population-based studies were conducted before ~~the~~ mass COVID-19 vaccination, and very few studies sampled populations in 2022 [12]. In Brazil, a nationwide household survey showed city-level prevalence had increased from 0% to 25.4% by June 2020, with a high heterogeneity among regions [13]. In the municipality of São Paulo, as part of the local public health response, sequential population-based household surveys estimated 9.7% SARS-CoV-2 seroprevalence in July 2020, with an increase to

25% by February 2021 [14]. SARS-CoV-2 had been difficult to control and still imposes challenging uncertainties, and serial population-based studies help understand the infection's complex and changing population dynamics.

The objectives of this study were to describe the SARS-CoV-2 seroprevalence estimates in the municipality of São Paulo and to explore the seroprevalence trends by sociodemographic characteristics, considering ~~the~~ pre- and post-vaccine implementation in the city. Lastly, we compared the overall seropositivity in the adult population with the COVID-19 cumulative registered cases during the pre-vaccination period.

Materials and Methods

Study design and sampling

The SoroEpi Study in~~at~~ the m~~M~~unicipality of São Paulo (SoroEpi MSP) comprised seven household-SARS-CoV-2 serosurveys from June 2020 to April 2022: Survey 1 (15-24 June 2020), Survey 2 (20-29 July 2020), Survey 3 (1-10 October 2020), Survey 4 (14-23 January 2021), Survey 5 (22 April-1 May 2021), Survey 6 (9-10 September 2021), and Survey 7 (31 Mar-9 April 2022). Figure 1 shows the number of households and adults by survey, which followed the study design.

Fig 1. Flow diagram for recruitment into the study by serosurvey, The SoroEpi MSP, Municipality of São Paulo, SP, Brazil, June 2020 to April 2022

Before initiating the full-scale surveys, a pilot study was conducted in May 2020, restricted to the six most COVID-19-affected districts in São Paulo. The data collection procedures proved to be adequate and feasible. The detailed methodology and preliminary seroprevalence of SARS-CoV-2 were published previously ~~published~~ [15]. To provide the COVID-19 local epidemiological context for each survey~~'s~~ time frame, secondary data were retrieved from the official sources of ~~as~~ the reported cumulative cases and deaths (Figure 2). We also retrieved SARS-CoV-2 variants circulating during the period of surveys in São Paulo state, traced by GISAID [16]. From June 2020 to January 2021 the predominant circulating variant was B1.1.28 with the entry of the Gamma variant of concern (VOC) at the beginning of ~~the year~~ 2021. The Gamma variant was the main circulating VOC by April 2021; Delta VOC by September 2021 and Omicron VOC by April 2022.

Fig 2. Timeline of COVID-19 cumulative cases and deaths according to seroprevalence surveys periods, The SoroEpi MSP Study, Municipality of São Paulo, Brazil, 2020-2022.

The surveys were designed following the World Health Organization (WHO) protocol for population-level COVID-19 antibody testing [17]. They were

cross-sectional population-based studies, and the target population was the adult (18 years old or older) inhabitants of permanent private households in the municipality of São Paulo.

According to the 2020 projections for the municipality of São Paulo by the Statewide System for Data Analysis Foundation (Fundação SEADE), 9,164,926 residents were 18 years of age and over out of an estimated total population of 11,869,660 [18]. Administratively, the municipality is organized into 96 districts. For this study, the adult population was divided into two strata of equal size using the average income of the municipality districts as the stratification variable. A sample size of 560 individuals was~~ere~~ planned in each stratum to allow the estimation of ~~the~~ seroprevalence greater than 4% to be obtained under coefficients of variation of less than 30%, considering a design effect of 2 (deff = 2).

We used a two-cluster probability sampling method to select the survey participants. In the first stage, we selected census tracts (115 in Survey 1 and 160 in the other six surveys) using probability proportional to the number of households counted in the 2010 census. In the second stage, we randomly selected households from each tract (12 in Survey 1 and eight in the subsequent surveys) from a list of households obtained by plotting out the tract. All adults in the selected households were eligible to participate in the study, except those with cognitive incapacities or health problems that make venipuncture difficult. Weights were introduced in the analysis to correct the different probabilities with which participants were randomly selected. These design weights were adjusted for non-response considering the response rates of three sets of census tracts

(ordered by average income, according to census data) and for the age and sex distribution observed in the city of São Paulo in the years 2020, 2021, and 2022.

Ethics and study procedures

Approval for the study protocol for the study was obtained from the National Research Ethics Commission (CONEP) and the Research Ethics Committee of Fleury Group (CAAE no 31032620.0.0000.5474). An amendment to the original serology survey was also approved by the Brazilian National Research Ethics Commission (CONEP) (CAAE no 31032620.0.0000.5474 v5) on January 14, 2021.

Formatted: Indent: First line: 0.49", Line spacing: Double

Formatted: Font: (Default) Calibri

Three visits were scheduled for each household on different days of the week and times of the day to limit the non-response. The sampling method did not permit replacements of the randomly selected sampling units, i.e., the census tracts and households. We recorded the frequency of households with eligible adults and the frequency of individuals interviewed with valid serological samples in each survey. We estimated the overall participation rate considering the number of included participants divided by the total number of individuals who would have been included according to the sampling frame, but who were considered non-participants because they were not reachable, or households could not be accessed. We assumed that the number of individuals per household was similar between the included households and the non-accessible selected households.

After participants signed~~ing~~ the written informed consent, face-to-face interviews were conducted using a questionnaire on mobile devices to obtain socio-demographic and other data. The information used in the present study comprised: s~~S~~ex, age, skin color/race and education measured by years of formal schooling~~ were the pieces of information used in the present study~~. Self-reported skin color/race categories followed the Brazilian Institute of Geography and Statistics (IBGE) classification: White, Black, mixed race (*P~~p~~ardo*), Asian, and Indigenous [19]. Questions about the date of vaccination, the number of doses, and the type of vaccine product were included in the questionnaire to account for the introduction of vaccination during Surveys 5, 6, and 7. The ~~Brazil's national~~ COVID-19 immunization program commenced nationally on 17 January 2021, and on the same date~~s well as~~ in São Paulo city (Figure 2). Four vaccines have been offered in the country's immunization programs: CoronaVac (Sinovac), an inactivated whole-virus COVID-19 vaccine, ChAdOx1 (AstraZeneca), Ad26.COV2.S (Janssen), and BNT162b2 (Pfizer-BioNTech).

Following the interview, about 3-5 ml of venous blood was collected from each participant, and samples were transported to the reference laboratory for antibody testing. The laboratory team had access to ~~the~~ information on~~f~~ individual participants during and after data collection to enable them to provide details of their results and any additional information the participants needed. ~~to allow them to learn about their results and contact the laboratory if they needed additional information.~~

Serology testing

Specimens were tested for anti-nucleocapsid total antibodies using the electrochemiluminescence Roche Elecsys Total Ig Assay (Roche Elecsys® Diagnostics, Rotkreuz, Switzerland) in Surveys 2 to 7. The sensitivity of the Ele~~y~~csys Anti-SARS-CoV-2 immunoassay is 99.5% (95% CI~~,~~ 97~~.0%~~-100~~.0%~~) and specificity 99.8% (95% CI~~,~~ 99.7~~%~~-99.9~~%~~), making it~~;~~ –a~~n~~ useful tool for determining ~~the~~ past SARS-CoV-2 infection, including at population level [20]~~, in Surveys 2 to 7~~. For Survey 1, the specimens were tested for immunoglobulin (Ig) M (IgM) and IgG using Maglumi SARS-CoV-2 chemiluminescence assay~~s~~ Immunoglobulin (Snibe Diagnostics, Shenzhen, China) [21] since that was the available test in the early months of the COVID-19 pandemic in 2020. ANVISA, the Brazilian Regulatory Health Agency, has cleared both assays for emergency use. Other tests were used in parallel with the Elecsys assay during the study period. However, for the sake of the present analyses, we opted to measure the anti-nucleocapsid total antibodies using ~~(~~Roche Elecsys~~)~~ due to its reported high sensitivity ~~(99.5%)~~ and specificity ~~(99.8%)~~ and because it has been broadly used in seroprevalence studies [20,22]. All tests were performed following the manufacturer's guidelines in the Grupo Fleury laboratory in São Paulo, Brazil.

Primary outcome

The primary outcome was seropositivity, which was defined according to previously set laboratory parameters. Individuals whose sera were reactive to the Maglumi assay in Survey 1 or Roche assay in Surveys 2 to 7 were considered positive. Indeterminate results were included as negative in the analyses. After the CoronaVac vaccine was introduced in early 2021, seropositivity could be

determined~~elicited~~ either by a viral infection or by vaccination and herein is defined as “hybrid immunity” [23].

Statistical analysis

Unadjusted and weighted frequency distributions were calculated with 95% confidence intervals (CI) by sex, age group, self-reported skin color, schooling, and mean income study design strata. Differences between categories were assessed using Rao-Scott chi-square tests. Estimates were obtained using the survey module of STATA version 14 (StataCorp, College Station, Texas, USA), in which the complex aspects of the sampling design were considered.

We applied joinpoint regression analysis to study the trends of sequential seroprevalence estimates of SARS-CoV-2 antibody surveys from June 2020 to April 2022 (seven surveys) using the JoinPoint app [24]. ~~This analytic approach allowed for identifying the time of the turning point(s) at which the trend significantly changes.~~ This model allowed us to test~~ing~~ whether the changes in the seroprevalence trend we~~a~~re statistically significant and to identify the time of the turning point ~~-~~separating the trend into distinct segments. Our dataset allowed us to choos~~eing~~ one as the maximum number of joinpoints in the~~a~~ linear model y = α + βx. The model selection used Monte Carlo permutation tests to determine the optimal number of joinpoints [25]. Each one performs a test of the null hypothesis H_0: 0 joinpoint against the alternative H_a: 1 joinpoint. This time series approach can distinguish different growth trends and their period (trend segments) according to the variables analysed. Therefore, the location of the

Formatted: Strikethrough

join~~t~~point (period) was based on statistical criteria resulting from the ~~as the result of jJ~~oin~~t~~p~~P~~oint calculations. ~~The location of the joinpoint was established based on statistical criteria.~~

Formatted: Strikethrough

Formatted: Strikethrough

Calculation of the reported fraction

We calculated the reported fraction as the ratio of the estimated number of infected individuals using primary seroprevalence data (Surveys 1 to 4) and the number of cumulative cases of COVID-19 extracted from the São Paulo Municipal Surveillance System. We considered the median time of the corresponding fieldwork period. The reported fractions were calculated for the surveys performed before the mass CoronaVac vaccination was introduced in São Paulo.

Results

For each survey, Figure 1 shows the number of randomly selected households (total n = 8,748) and the number of households identified with eligible adults (total n = 3,904), after excluding ~~the~~ households which were closed during the data collection periods (total n = 1,436) and those that preferred not to give ~~denied giving~~ any information (total n = 3,408). Overall, 44.6% of the randomly selected households were included in the study.

The seven consecutive surveys included 8,134 participants with complete interviews and blood specimens, which corresponded to 73.9% of the 11,013 eligible adults. However, if we consider the participation rate of the selected households together with the participation rate of the eligible adults, the overall participation rate was 38.4% (95% CI 37.7-39.1).

For each survey, the percentages of participants by sex, age group, and mean income study design strata agreed with the percentages of the population of the municipality of São Paulo after the sampling design weights were adjusted for these variables. Regarding race, approximately 50% of the participants self-declared as White, followed by approximately 42% as Black or mixed race (*P~~p~~ardo*), and less than 5% as Asian or Indigenous. The percentage of participants with 11 years or less of schooling ranged from 32.9% (Survey 2) to 39.5% (Survey 7) (S1 Table).

~~By April 2021 (Survey 5), 83.7% (95% CI: 81.1%-85.9%) of the São Paulo city population were unvaccinated, and 10.5% (95% CI: 8.5%-12.9%) had received at least one dose of CoronaVac. There was a sharp decline in the percentage of unvaccinated adults to 4.0% (95% CI: 2.8%-5.8%) in September 2021 (Survey 6) and 1.8% (95% CI: 0.9%-3.5%) by April 2022 (Survey 7). The CoronaVac vaccine (one or more doses) was predominantly administered in the oldest age group (60 years or older) and the 18-39 years group (S2 Table).~~

From June 2020 (Survey 1) to January 2021 (Survey 4), before mass vaccination, the weighted overall seroprevalence increased from 11.4% (95% CI: 9.2~~%~~-13.6~~%~~) to 24.9% (95% CI: 21.0~~%~~-28.7~~%~~). From April 2021 (Survey 5) to April 2022 (Survey 7), the seropositivity increased from 38.1% (95% CI: 34.3~~%~~-

41.9~~%~~) to 77.7% (95% CI: 74.4~~%~~-81.0~~%~~). Residents in the lower-income stratum had a higher weighted prevalence of SARS-CoV-2 antibodies than ~~compared to~~ the high-income stratum in all surveys except for Survey 2. None of the surveys showed a significant difference in the frequency of seropositivity between women and men. Among the oldest age group, the prevalence ranged from 11.1% (95% CI: 6.0~~%~~-16.0~~%~~) in Survey 1 to 65.4% (95% CI: 58.1~~%~~-72.6~~%~~) in Survey 7, while in the youngest age group, it varied from 10% (95% CI: 7.1~~%~~-12.8~~%~~) to 85.8% (95% CI: 81.6~~%~~-90.0~~%~~). The self-declared Black or mixed race (*P~~p~~ardo*) group presented statistically higher seroprevalence in all surveys when compared to the White group. The population with 16 or more years of education had lower seroprevalence, in all except in the last two surveys (Table 1).

In~~By~~ April 2021 (Survey 5), 83.7% (95% CI: 81.1~~%~~-85.9~~%~~) of the São Paulo city population were unvaccinated, and 10.5% (95% CI: 8.5~~%~~-12.9~~%~~) had received at least one dose of CoronaVac. There was a sharp decline in the percentage of unvaccinated adults to 4.0% (95% CI: 2.8~~%~~-5.8~~%~~) in September 2021 (Survey 6) and 1.8% (95% CI: 0.9~~%~~-3.5~~%~~) by April 2022 (Survey 7). The CoronaVac vaccine (one or more doses) was the most commonly used ~~predominantly administered~~ in the oldest age group (60 years or older) and the 18-39 years group (S2 Table).

Formatted: Not Highlight

Table 1. Weighted seroprevalence estimates (and 95% CI) of anti-SARS-CoV-2 per survey, The SoroEpi MSP Study, Municipality of São Paulo, SP, Brazil, June 2020 to April 2022.

Characteristic	Survey 1[#] June 2020 n	Survey 1[#] June 2020 % 95% CI	Survey 2 July 2020 n	Survey 2 July 2020 % 95% CI	Survey 3 October 2020 n	Survey 3 October 2020 % 95% CI	Survey 4 January 2021 n	Survey 4 January 2021 % 95% CI	Survey 5 April 2021 n	Survey 5 April 2021 % 95% CI	Survey 6 September 2021 n	Survey 6 September 2021 % 95% CI	Survey 7 April 2022 n	Survey 7 April 2022 % 95% CI
**Overall**	1183	11.4 (9.2-13.6)	1470	14.8 (12.1-17.6)	1129	18.6 (15.2-22.0)	1194	24.9 (21.0-28.7)	1187	38.1 (34.3-41.9)	1035	52.7 (48.7-56.8)	936	77.7 (74.4-81.0)
**Study strata (mean income)**	p<0.0001		p=0.2072		p=0.0083		p=0.0004		p=0.0022		p=0.0001		p<0.0001	
High	676	6.5 (4.4-8.5)	851	13.0 (9.2-16.8)	544	13.9 (9.6-18.2)	580	17.6 (12.7-22.6)	610	32.0 (26.7-37.4)	415	44.9 (39.2-50.5)	435	70.7 (65.1-76.2)
Low	507	16.0 (12.2-19.8)	619	16.5 (12.6-20.4)	585	23.0 (18.0-28.0)	614	31.9 (26.1-37.6)	577	44.0 (38.8-49.2)	620	60.4 (55.3-65.4)	501	84.2 (80.8-87.6)
**Sex**	p=0.8192		p=0.7207		p=0.6261		p=0.8207		p=0.3694		p=0.6701		p=0.8619	
Male	521	11.6 (8.7-14.5)	607	14.5 (10.9-18.0)	460	18.0 (13.2-22.8)	483	24.6 (19.4-29.7)	481	36.7 (31.7-41.7)	368	53.7 (46.9-60.5)	303	78.0 (73.3-82.6)
Female	662	11.2 (8.5-13.9)	863	15.1 (12.0-18.3)	669	19.2 (15.7-22.6)	711	25.1 (21.2-29.1)	706	39.4 (34.7-44.0)	667	51.9 (47.0-56.8)	633	77.5 (73.5-81.5)
**Age group (years)**	p=0.3585		p=0.0850		p=0.0237		p<0.0001		p=0.0001		p=0.0087		p<0.0001	
18 - 39	496	10.0 (7.1-12.8)	613	15.0 (11.7-18.4)	459	21.8 (17.2-26.4)	497	29.4 (24.4-34.3)	502	41.6 (36.3-47.0)	414	58.8 (53.3-64.3)	325	85.8 (81.6-90.0)
40 - 59	435	13.4 (9.7-17.1)	494	16.8 (12.3-21.2)	397	18.2 (13.2-23.3)	425	25.6 (20.6-30.6)	462	40.7 (35.6-45.8)	357	48.1 (41.7-54.4)	360	75.3 (69.7-80.9)
≥ 60	252	11.1 (6.0-16.2)	363	10.9 (7.4-14.5)	273	12.3 (7.5-17.2)	272	14.1 (9.3-19.0)	223	26.3 (20.5-32.1)	264	47.9 (40.7-55.1)	251	65.4 (58.1-72.6)
**Self-reported race/skin color**	p=0.0055		p=0.0543		p=0.0012		p<0.0001		p<0.0001		p=0.0011		p=0.0051	
White	614	7.9 (5.6-10.4)	733	12.9 (9.6-16.2)	556	14.4 (10.6-18.2)	609	18.0 (13.9-22.0)	537	30.9 (25.9-35.9)	406	45.3 (39.5-51.1)	435	72.1 (67.1-77.0)
Black or mixed (*Pardo*)	510	15.3 (11.9-18.7)	671	17.0 (13.2-20.7)	530	23.4 (19.0-27.8)	549	33.3 (27.9-38.7)	584	45.5 (40.8-50.2)	599	58.6 (53.9-63.3)	470	82.8 (79.0-86.5)
Asian or Indigenous	48	9.7 (0.0-20.6)	49	8.5 (1.6-15.4)	33	11.8 (0.6-23.0)	30	9.3 (0.0-19.9)	58	28.7 (15.9-41.5)	25	45.5 (21.6-69.3)	24	80.4 (60.5-100)
**Schooling (years)**	p=0.0004		p=0.0007		p=0.0001		p<0.0001		p<0.0001		p=0.0231		p=0.0469	
≥ 11	386	17.2 (12.2-22.2)	518	17.8 (13.9-21.7)	429	24.5 (18.7-30.3)	442	29.7 (23.6-35.9)	415	41.2 (35.4-47.0)	417	55.1 (49.9-60.3)	370	80.6 (76.5-84.7)
12 - 15	464	11.0 (8.4-13.5)	586	16.0 (12.2-19.8)	426	19.0 (14.6-23.4)	474	27.2 (22.7-31.7)	483	46.1 (40.7-51.6)	416	56.4 (51.1-61.7)	381	78.7 (73.8-83.6)
≥ 16	333	5.1 (1.8-8.4)	366	8.9 (5.9-11.9)	274	9.7 (6.0-13.4)	278	14.3 (9.7-18.9)	289	21.5 (15.6-27.4)	202	42.8 (33.0-52.6)	185	70.4 (62.5-78.3)

#Survey 1, the specimens were tested for IgM and IgG with the Maglumi SARS-CoV-2 test.
In Surveys 2 to Survey 7, the specimens were tested for anti-nucleocapsid total antibodies with the Roche Elecsys test.

In all surveys, seroprevalence estimates were weighted by sampling design with adjustments for non-response.

Table 2 shows the growth models’ parameters for ~~the~~ prevalence stratified by sex, age group and self-reported race/skin color, and Figure 3 presents the observed and modelled prevalence data for each of these variables. The overall trend of the point prevalence series (Figure 3a) identified two periods with significantly different growths: a monthly increase of approximately 2% (β1) in prevalence from June 2020 (Survey 1) to January 2021 (Survey 4) and a monthly increase of approximately 3.5% (β2) in prevalence from January 2021 (Survey 4) to April 2022 (Survey 7). For both sexes (Figure 3b), there is a joinpoint in January 2021 (Survey 4), and the overlap of the prevalence curves in the whole study period illustrates the lack of difference between the two groups. The trend analysis of the prevalence by age group (Figure 3c) indicated one joinpoint in January 2021 (Survey 4) for the 18-39 and 60 or older groups. After this point, both groups presented similar monthly increases (β2 = 3.5% and 3.7%, respectively), although the prevalence magnitudes differed. For the White self-reported race group, the set identified one joinpoint in January 2021 (Survey 4) and a significant increase of 3.5% between January 2021 and April 2022,~~.~~ w~~W~~hereas the Black or mixed self-reported race group showed a significant monthly increase of 3.1% over~~in~~ the whole study period (Figure 3d).

Table 2. Growth models and parameter estimates of point prevalence of SARS-CoV-2 antibody encompassing seven population-based surveys, The SoroEpi MSP Study, Municipality of São Paulo, SP, Brazil, June 2020 to April 2022.

	Period	Parameter	Estimate	Standard Error	Test Statistic (t)	Prob > |t|
**Overall**						
	Jun 2020 - Jan 2021	α1	11.785	0.838	14.065	0.005
		β1	1.987*	0.445	4.463	0.047
	Jan 2021 - Apr 2022	α2	1.404	3.130	0.448	0.698
		β2	3.470*	0.183	18.958	0.003
**Study strata (mean income)**						
High	Jun 2020 - Apr 2022	α1	6.093	1.686	3.614	0.015
		β1	2.702*	0.221	12.239	0.000
Low	Jun 2020 - Oct 2020	α1	15.475	0.614	25.217	0.002
		β1	1.792	0.877	2.043	0.178
	Oct 2020 – Apr 2022	α2	8.947	1.050	8.518	0.014
		β2	3.424*	0.062	55.399	0.000
**Sex**						
Male	Jun 2020 - Jan 2021	α1	11.849	0.519	22.849	0.002
		β1	1.883*	0.294	6.415	0.023
	Jan 2021- Apr 2022	α2	0.210	1.999	0.105	0.926
		β2	3.546*	0.117	30.191	0.001
Female	Jun 2020 - Jan 2021	α1	11.815	1.229	9.611	0.011
		β1	2.056	0.586	3.508	0.073
	Jan 2021 - Apr 2022	α2	2.337	4.713	0.496	0.669
		β2	3.410*	0.273	12.469	0.006
**Age group (years)**						
18-39	Jun 2020 - Jan 2021	α1	10.895	0.931	11.704	0.007
		β1	2.726*	0.522	5.219	0.035
	Jan 2021 - Apr 2022	α2	4.006	3.718	1.078	0.394
		β2	3.710*	0.212	17.502	0.003
40-59	Jun 2020 - Apr 2022	α1	11.563	1.998	5.789	0.002
		β1	2.725*	0.208	13.087	0.000
≥ 60	Jun 2020 - Jan 2021	α1	10.458	1.593	6.563	0.022
		β1	0.652	0.733	0.890	0.467
	Jan 2021 - Apr 2022	α2	-9.737	5.423	-1.795	0.214
		β2	3.537*	0.348	10.170	0.010

*Slope significantly different from zero at the alpha=0.05 level.

Fig 3. Joinpoint trends for SARS-CoV-2 seropositivity in the Municipality of São Paulo according to surveys from June 2020 to April 2022. a) overall, b) sex, c) age group, and d) self-reported race/skin color.

Table 3 shows the estimated population who had had SARS-CoV-2 infection in the pre-vaccination period (Surveys 1 to 4) in the municipality of São Paulo, the number of cumulative reported COVID-19 cases and the infection-to-case ratio. The estimated number of persons who were~~had an~~ infected~~ion~~ ranged from 1.1 million in June 2020 (Survey 1) to approximately 2.3 million in January 2021 (Survey 4). The underreporting multiplier varied from 8.9 in Survey 1 to 4.3 in Survey 4.

Table 3. Estimated number of adults infected with SARS-CoV-2 and the ratio of estimated infections by reported COVID-19 cases in the pre-vaccination period, The SoroEpi MSP Study, Municipality of São Paulo, SP, Brazil, June 2020 to January 2021.

Survey (specimen collection period)	Population*	Sample size n	SARS-CoV-2 seroprevalence % (95% CI)	Estimated number of adults with SARS-CoV-2 infection n (95% CI)	Cumulative reported COVID-19 cases (date)**	Ratio (infections:cases)
Survey 1 (15-24 June 2020)	9,164,926	1,183	11.4 (9.2-13.6)	1,044,802 (843,173-1,246,430)	117,768 (20 June)	8.9 (7.2-10.6)
Survey 2 (20-29 July 2020)	9,164,926	1,470	14.8 (12.1-17.6)	1,356,409 (1,108,956-1,613,027)	203,308 (24 July)	6.7 (5.5-7.9)
Survey 3 (1-10 October 2020)	9,164,926	1,129	18.6 (15.2-22.0)	1,704,676 (1,393,069-2,016,284)	337,206 (5 October)	5.1 (4.1-6.0)
Survey 4 (14-23 January 2021)	9,216,158	1,194	24.9 (21.0-28.7)	2,294,823 (1,935,393-2,645,037)	528,809 (18 January)	4.3 (3.7-5.0)

*Population aged 18 years and older projected by Fundação SEADE for the m~~m~~unicipality of São Paulo.

**Source: Secretaria Municipal de Saúde, São Paulo.

Discussion

The present study presentsed new data on the seroprevalence trends of SARS-CoV-2 antibodies in the general adult population ofin the municipality of São Paulo, Brazil, based on seven cross-sectional surveys over an almost 2-year period. Our data showed that SARS-CoV-2 seropositivity increased over time, ranging from 11.4% (95%CI: 9.2-13.6) in June 2020 to 77.7% (95%CI: 74.4-81.0) in April 2022. We observed a significantly higher increase in the seroprevalence trend from beginning in January 2021 up to April 2022 compared to 2020, most probably driven by the implementation of the COVID-19 vaccinatione campaigns.

Our results revealed that approximately 80% of the adult population in the municipality of São Paulo was susceptible to SARS-CoV-2 infection before vaccine availability in January 2021. A relatively small variation, from 11.4% (95%CI: 9.2-13.6) in June 2020 to 18.6% (95%CI: 15.2-22.0) in October 2020, measured natural immunity using an anti-nucleocapsid antibody test.- -In the pre-COVID-19 vaccine era, serosurveys using rapid tests (lateral flow Wondfo Biotech) in the city of São Paulo showed an even lower seroprevalence of SARS-CoV-2 (13.6%) by September 2020 [14]. Buss et al reported 44% of prevalence of IgG antibodies to SARS-CoV-2 nucleocapsid (N) protein (Abbot), among blood donors in the city of Manaus (Brazilian Amazon) by June 2020. The prevalence

rate was higher in the Amazon tha~~e~~n in São Paulo (13.6%,~~;~~ 95% CI: 12.0~~%~~-18.1~~%~~) in the same period, showing distinct patterns of the epidemic within the country [26]. The prevalence reported among blood donors is similar to the~~of~~ 11.4% (95% CI: 9.2~~%~~-13.6~~%~~) found ~~conducted~~ in the general population in~~by~~ June 2020 (Survey-1). A serial cross-sectional SARS-CoV-2 antibody (Abbot assay) among blood donors showed a range of adjusted seroprevalence from 19.0% (95% CI 15.9-22.4) in~~by~~ December 2020 to 22.3% (95%CI19.0-25.9) in~~by~~ February 2021 in the m~~m~~unicipality of São Paulo [27]. These findings are in line with our population-based level of 24.9% (95% CI 21.0-28.7) of weighted seroprevalence of anti-SARS-CoV-2 in~~by~~ January 2021 (Survey-4), which is within ~~in between~~ the time period analysed by Prete et al ~~(,).~~. However, comparison may be hampered by differences in the assays performed~~ance~~ and the study populations ~~selected~~. A systematic review and pooled analysis estimated that around 10% of the Brazilian population had been infected by March 2021 [28]. Globally, there was a large variation in prevalence among the geographic regions over time, and the authors called attention to the need for continuous monitoring of the population's immunity levels [12,28,29].

The high level of seropositivity in the adult population was only reached by the date of our last survey (April 2022). This population-level immunity reflected the past virus infection and/or vaccination, mostly with CoronaVac, eliciting anti-nucleocapsid antibodies. The high level of hybrid immunity was mainly achieved by extensive vaccination coverage. We acknowledge that the reported positivity of SARS-CoV-2 antibodies at the population level did not include the anti-Spike antibodies elicited by other vaccine types [30]. In this sense, we may assume that the overall seroprevalence ~~estimated~~ in our study could~~may~~ be underestimated.

A seroprevalence of more than 95%, using SARS-CoV-2 anti-Spike protein assay, was reported among blood donors in the city of São Paulo and six other Brazilian capitals in~~by~~ November 2021 [31]. Our results were similar to those observed in serial surveys among blood donors in the US, where the seroprevalence induced by hybrid immunity reached more than 80% [32]. In our study, adults living in districts classified as low-income stratum, and those who self-declared Black or mixed (*P~~p~~ardo)*, were the most infected over the entire study period, in line with other serosurveys conducted in Brazil [14,33] and elsewhere [28]. In our setting, during 2020, higher COVID-19 mortality was observed among the lower-income stratum and Black populations, showing marked social inequalities [34,35].

In our models, the upward trend of the overall seroprevalence and the seroprevalence estimates stratified by sex, age group and self-reported race, indicated that January 2021 was the inflection point in the study period. The overlapping of the growth curves for males and females suggested similarities in the frequency of SARS-CoV-2 viral infection as the result of natural immunity in the year 2020 and/or vaccine-induced immunity afterwards. People over~~lder than~~ 60 years of age showed low seroprevalence, around 10%, with no significant increase in the year 2020, in contrast to~~with~~ significant increases among the younger age groups. This likely reflected the higher compliance to lockdown or social distance measures among the older population reported in other Brazilian regions [36]. Considering the fitted models for those aged 60 years or older, there was around a threefold increase in the slope estimated after January 2021. This high slope in prevalence growth occurred concurrently with the prioritization of COVID-19 vaccination~~e~~ ~~implementation~~ among the older population. Therefore,

vaccination seems to be the likely explanation for the rapid increase in hybrid immunity in older individuals. Of note, approximately half of the older population received the CoronaVac vaccine, the first vaccine product available in Brazil.

We found at least 8.9 (June 2020) and 4.3 (January 2021) times higher numbers of SARS-CoV-2 infections than the number of COVID-19 cases reported by the official surveillance system in 2020. These figures aligned with the 6.9 overall estimated ratio of the SARS-CoV-2 infection by the confirmed COVID-19 reported cases in the region of the Americas, based mainly on ~~the~~ US serosurveys [37]. Several possible explanations could be offered ~~raised~~ for the variation of the infections:reported cases ratio in our setting. Asymptomatic or mild symptomatic cases were seldom reported; laboratory tests for disease confirmation were restricted to severe cases in the early phases of the epidemic; and there were changes in the criteria for reporting COVID-19 cases, with cases being confirmed based on symptoms and imaging tests [38].

Population-based seroprevalence surveys of SARS-CoV-2 can provide estimates of the magnitude of SARS-CoV-2 infections irrespective of case-based surveillance [39]. In the UK, the Office for~~ial~~ National Statistics has been performing repeated antibody~~ies~~ and RT-PCR swab surveys since 2020, in addition to gathering ~~the~~ case-surveillance data [40]. Also, in the US, a large national probabilistic repeated household survey has been in place to estimate the cumulative incidence in the stages of the COVID-19 pandemic [41].

SARS-CoV-2 was~~had been~~ difficult to control and still imposes challenging uncertainties [42]. Sequential population-based studies with probabilistic

sampling and highly accurate laboratory assays are central to~~for~~ monitoring the infection’s complex and changing population dynamics.

To our knowledge, this was the only seven-round study of the point prevalence of SARS-CoV-2 antibodies in a representative sample of the general adult population of the municipality of São Paulo. It included the pre- and post-vaccination periods throughout the two years of the pandemic. A key strength of our study was the use of a robust methodology including the probability sampling method to select the survey participants, together with statistical weighting and adjustments for demographics and non-response. The use of a highly sensitive, specific and fully automated assay to detect anti-nucleocapsid SARS-CoV-2 immunoglobulins was another strength. Moreover, the same rigorous methodology, data analysis strategies, and testing procedures were repeated in all surveys, despite the adverse conditions such as periods ~~times~~ of lockdown and social distancing.

Although our study has significant strengths, several limitations must be acknowledged. The first limitation was that we did not include people under 18 years old due to logistic, time, and cost restrictions. Second, like most population-based household serosurveys [12], our study had a low household response rate. Third, we used an anti-SARS-CoV-2-nucleocapsid test that did not distinguish between antibodies induced by natural infection and those induced by vaccines. Fourth, seroprevalence results in Survey 1 were derived from the available laboratory test at the time, which was not the same as the test used in Surveys 2-7. Since there was only a one-month interval between Survey 1 and Survey 2, and both yielded similar estimates, we judged that it did not introduce measurement bias. Fifth, we did not consider the antibody waning over the

months following infection, which could have led tocaused underestimated results. -However,In addition, we used Roche Elecsys immunoassay which that is considered a high sensitivity, high specificity assay and less prone to antibody waning [43]. Our study design which consisted of seven independent population-based surveys dido not allow us to measure antibody waning directly as in the cohort studies.

Last, it is important to recognize that the joinpoint trend analysis is usually performed in longer series of point estimates composed of higher numbers of observations.

Formatted: Font: (Default) Calibri, 11 pt

Conclusion

Our results documented the evolution of the SARS-CoV-2 infection patterns and vaccine response throughout the two years of the pandemic in the municipality of São Paulo. It made it possenabled usible to learn about the epidemiological features of the infection, evidencing the complexity of the population-based seropositivity profile. Our study reinforced the importance of conducting repeated serological surveillance to monitor and study how new infectious pathogen transmission dynamics evolve in susceptible populations. Such data may add to the understanding of the complex and changing population dynamics of the SARS-CoV-2 infection, including the impact of vaccination strategies and the modelling of future epidemiological scenarios.

Acknowledgments

We thank all the study participants whose cooperation made the study possible.

References

1. Castro MC, Kim S, Barberia L, Ribeiro AF, Gurzenda S, Ribeiro KB, et al. Spatiotemporal pattern of COVID-19 spread in Brazil. Science. 2021;372: 821–826. doi:10.1126/science.abh1558
2. WHO Health Emergency Dashboard. WHO Coronavirus (COVID-19) Dashboard. [cited 8 Nov 2022]. Available: https://covid19.who.int
3. WHO Health Emergency Dashboard. Brazil: WHO Coronavirus Disease (COVID-19) Dashboard With Vaccination Data. [cited 8 Nov 2022]. Available: https://covid19.who.int/region/amro/country/br
4. Worldometer. Brazil Population (2022). [cited 8 Nov 2022]. Available: https://www.worldometers.info/world-population/brazil-population/
5. Góes C, Karpowicz I. Inequality in Brazil: A Regional Perspective. 2017;2017: 34. doi:https://doi.org/10.5089/9781484324776.001
6. IBGE. Instituto Brasileiro de Geografia e Estatística. Projeções da População IBGE. [cited 7 Nov 2022]. Available: https://www.ibge.gov.br/estatisticas/sociais/populacao/9109-projecao-da-populacao.html?=&t=resultados
7. Candido DDS, Watts A, Abade L, Kraemer MUG, Pybus OG, Croda J, et al. Routes for COVID-19 importation in Brazil. J Travel Med. 2020;27: taaa042. doi:10.1093/jtm/taaa042
8. Candido DS, Claro IM, de Jesus JG, Souza WM, Moreira FRR, Dellicour S, et al. Evolution and epidemic spread of SARS-CoV-2 in Brazil. Science.

2020;369: 1255–1260. doi:10.1126/science.abd2161

9. Secretaria Municipal da Saúde. Prefeitura da Cidade de São Paulo. Boletim Semanal Covid-19 no município de São Paulo de 14 de setembro de 2022. Prefeitura da Cidade de São Paulo; 14 Sep 2022 [cited 21 Dec 2022]. Available: https://www.prefeitura.sp.gov.br/cidade/secretarias/saude/vigilancia_em_saude/doencas_e_agravos/coronavirus/index.php?p=335024
10. Bryant JE, Azman AS, Ferrari MJ, Arnold BF, Boni MF, Boum Y, et al. Serology for SARS-CoV-2: Apprehensions, opportunities, and the path forward. Sci Immunol. 2020;5: eabc6347. doi:10.1126/sciimmunol.abc6347
11. Murhekar MV, Bhatnagar T, Selvaraju S, Saravanakumar V, Thangaraj JWV, Shah N, et al. SARS-CoV-2 antibody seroprevalence in India, August–September, 2020: findings from the second nationwide household serosurvey. Lancet Glob Health. 2021;9: e257–e266. doi:10.1016/S2214-109X(20)30544-1
12. Bergeri I, Whelan MG, Ware H, Subissi L, Nardone A, Lewis HC, et al. Global SARS-CoV-2 seroprevalence from January 2020 to April 2022: A systematic review and meta-analysis of standardized population-based studies. PLOS Med. 2022;19: e1004107. doi:10.1371/journal.pmed.1004107
13. Hallal PC, Hartwig FP, Horta BL, Silveira MF, Struchiner CJ, Vidaletti LP, et al. SARS-CoV-2 antibody prevalence in Brazil: results from two successive nationwide serological household surveys. Lancet Glob Health. 2020;8: e1390–e1398. doi:10.1016/S2214-109X(20)30387-9
14. Albuquerque JOM de, Kamioka GA, Madalosso G, Costa SA, Ferreira PB, Pino FA, et al. Prevalence evolution of SARS-CoV-2 infection in the city of São Paulo, 2020–2021. Rev Saúde Pública. 2021;55: 62–62. doi:10.11606/s1518-8787.2021055003970
15. Tess BH, Granato CFH, Alves MCGP, Pintão MCT, Nunes MC, Rizzatti EG, et al. Assessment of initial SARS-CoV-2 seroprevalence in the most affected districts in the municipality of São Paulo, Brazil. Braz J Infect Dis. 2021;25. doi:10.1016/j.bjid.2021.101604
16. FIOCRUZ. FIOCRUZ: Genomic Network. In: Genomahcov - Fiocruz [Internet]. [cited 9 Feb 2023]. Available:

Formatted: Portuguese (Brazil)

Field Code Changed

Formatted: French (France)

Field Code Changed

Formatted: Portuguese (Brazil)

https://www.genomahcov.fiocruz.br/dashboard-pt/

17. WHO. Population-based age-stratified seroepidemiological investigation protocol for coronavirus 2019 (COVID-19) infection. World Health Organ. 2020; 20.
18. SEADE. Fundação Sistema Estadual de Análise de Dados. Sistema SEADE de Projeções Populacionais. [cited 7 Nov 2022]. Available: http://produtos.seade.gov.br/produtos/projpop/
19. IBGE. Instituto Brasileiro de Geografia e Estatística. Desigualdades sociais por cor ou raça no Brasil. [cited 20 Dec 2022]. Available: https://biblioteca.ibge.gov.br/index.php/biblioteca-catalogo?view=detalhes&id=2101972
20. Muench P, Jochum S, Wenderoth V, Ofenloch-Haehnle B, Hombach M, Strobl M, et al. Development and validation of the Elecsys Anti-SARS-CoV-2 immunoassay as a highly specific tool for determining past exposure to SARS-CoV-2. J Clin Microbiol. 2020;58.
21. Padoan A, Cosma C, Sciacovelli L, Faggian D, Plebani M. Analytical performances of a chemiluminescence immunoassay for SARS-CoV-2 IgM/IgG and antibody kinetics. Clin Chem Lab Med. 2020;58: 1081–1088. doi:10.1515/cclm-2020-0443
22. Peluso MJ, Takahashi S, Hakim J, Kelly JD, Torres L, Iyer NS, et al. SARS-CoV-2 antibody magnitude and detectability are driven by disease severity, timing, and assay. Sci Adv. 2021;7: eabh3409. doi:10.1126/sciadv.abh3409
23. WHO. Interim statement on hybrid immunity and increasing population seroprevalence rates. 1 Jun 2022 [cited 21 Dec 2022]. Available: https://www.who.int/news/item/01-06-2022-interim-statement-on-hybrid-immunity-and-increasing-population-seroprevalence-rates
24. Statistical Methodology and Applications Branch, Surveillance Research Program, National Cancer Institute. Joinpoint Regression Program. 2022. Available: https://surveillance.cancer.gov/joinpoint/
25. Kim HJ, Fay MP, Feuer EJ, Midthune DN. Permutation tests for joinpoint regression with applications to cancer rates. Stat Med. 2000;19: 335–351. doi:10.1002/(sici)1097-0258(20000215)19:3<335::aid-sim336>3.0.co;2-z
26. Buss LF, Prete CA, Abrahim CMM, Mendrone A, Salomon T, de Almeida-

Formatted: Portuguese (Brazil)
Formatted: Portuguese (Brazil)
Formatted: Portuguese (Brazil)
Field Code Changed
Formatted: Portuguese (Brazil)
Formatted: Portuguese (Brazil)
Formatted: Portuguese (Brazil)
Field Code Changed
Formatted: Portuguese (Brazil)
Formatted: Portuguese (Brazil)

Neto C, et al. Three-quarters attack rate of SARS-CoV-2 in the Brazilian Amazon during a largely unmitigated epidemic. Science. 2021;371: 288–292. doi:10.1126/science.abe9728
27. Prete CA, Buss LF, Whittaker C, Salomon T, Oikawa MK, Pereira RHM, et al. SARS-CoV-2 antibody dynamics in blood donors and COVID-19 epidemiology in eight Brazilian state capitals: A serial cross-sectional study. eLife. 2022;11: e78233. doi:10.7554/eLife.78233
28. Metzger C, Leroy T, Bochnakian A, Jeulin H, Gegout-Petit A, Legrand K, et al. Seroprevalence and SARS-CoV-2 invasion in general populations: A scoping review over the first year of the pandemic. PLOS ONE. 2023;18: e0269104. doi:10.1371/journal.pone.0269104
29. Rostami A, Sepidarkish M, Fazlzadeh A, Mokdad AH, Sattarnezhad A, Esfandyari S, et al. Update on SARS-CoV-2 seroprevalence: regional and worldwide. Clin Microbiol Infect. 2021;27: 1762–1771. doi:10.1016/j.cmi.2021.09.019
30. Duarte N, Yanes-Lane M, Arora RK, Bobrovitz N, Liu M, Bego MG, et al. Adapting Serosurveys for the SARS-CoV-2 Vaccine Era. Open Forum Infect Dis. 2022;9: ofab632. doi:10.1093/ofid/ofab632
31. Buss L, Prete CA, Whittaker C, Salomon T, Oikawa MK, Pereira RHM, et al. Predicting SARS-CoV-2 Variant Spread in a Completely Seropositive Population Using Semi-Quantitative Antibody Measurements in Blood Donors. Vaccines. 2022;10: 1437. doi:10.3390/vaccines10091437
32. Jones JM, Stone M, Sulaeman H, Fink RV, Dave H, Levy ME, et al. Estimated US Infection- and Vaccine-Induced SARS-CoV-2 Seroprevalence Based on Blood Donations, July 2020-May 2021. JAMA. 2021;326: 1400–1409. doi:10.1001/jama.2021.15161
33. Barros AJD, Victora CG, Menezes AMB, Horta BL, Barros FC, Hartwig FP, et al. Population-level seropositivity trend for SARS-Cov-2 in Rio Grande do Sul, Brazil. Rev Saúde Pública. 2021;55. doi:10.11606/s1518-8787.2021055004075
34. Ribeiro KB, Ribeiro AF, Veras MA de SM, de Castro MC. Social inequalities and COVID-19 mortality in the city of São Paulo, Brazil. Int J Epidemiol. 2021;50: 732–742. doi:10.1093/ije/dyab022
35. Li SL, Pereira RHM, Jr CAP, Zarebski AE, Emanuel L, Alves PJH, et al.

Formatted: French (France)
Formatted: French (France)
Formatted: French (France)
Field Code Changed
Formatted: Portuguese (Brazil)
Formatted: Portuguese (Brazil)
Formatted: Portuguese (Brazil)
Field Code Changed
Formatted: Portuguese (Brazil)
Formatted: Portuguese (Brazil)
Formatted: Portuguese (Brazil)
Field Code Changed
Formatted: Portuguese (Brazil)
Formatted: Portuguese (Brazil)

Higher risk of death from COVID-19 in low-income and non-White populations of São Paulo, Brazil. BMJ Glob Health. 2021;6: e004959. doi:10.1136/bmjgh-2021-004959

36. Carvalho MS, Bastos LS, Fuller T, Cruz OG, Damasceno L, Calvet G, et al. Incidence of SARS-CoV-2 over four epidemic waves in a low-resource community in Rio de Janeiro, Brazil: A prospective cohort study. Lancet Reg Health – Am. 2022;12. doi:10.1016/j.lana.2022.100283

37. Chen X, Chen Z, Azman AS, Deng X, Sun R, Zhao Z, et al. Serological evidence of human infection with SARS-CoV-2: a systematic review and meta-analysis. Lancet Glob Health. 2021;9: e598–e609. doi:10.1016/S2214-109X(21)00026-7

38. Barberia LG, Cantarelli LGR, Oliveira MLC de F, Moreira N de P, Rosa ISC. The effect of state-level social distancing policy stringency on mobility in the states of Brazil. Rev Adm Pública. 2021;55: 27–49. doi:10.1590/0034-761220200549

39. Shook-Sa BE, Boyce RM, Aiello AE. Estimation Without Representation: Early Severe Acute Respiratory Syndrome Coronavirus 2 Seroprevalence Studies and the Path Forward. J Infect Dis. 2020;222: 1086–1089. doi:10.1093/infdis/jiaa429

40. Office for National Statistics (ONS). Coronavirus (COVID-19) Infection Survey, antibody data, UK: 5 October 2022. ONS website; 2022 Oct. Available: https://www.ons.gov.uk/peoplepopulationandcommunity/healthandsocialcare/conditionsanddiseases/bulletins/coronaviruscovid19infectionsurveyantibodyandvaccinationdatafortheuk/5october2022

41. National Institutes of Health. NIH begins study to quantify undetected cases of coronavirus infection. In: National Institutes of Health (NIH) [Internet]. 10 Apr 2020 [cited 7 Nov 2022]. Available: https://www.nih.gov/news-events/news-releases/nih-begins-study-quantify-undetected-cases-coronavirus-infection

42. Anderson RM, Vegvari C, Hollingsworth TD, Pi L, Maddren R, Ng CW, et al. The SARS-CoV-2 pandemic: remaining uncertainties in our understanding of the epidemiology and transmission dynamics of the virus, and challenges to be overcome. Interface Focus. 11: 20210008.

Formatted: Portuguese (Brazil)

Formatted: German (Germany)

Formatted: Portuguese (Brazil)

Formatted: French (France)

Formatted: French (France)

doi:10.1098/rsfs.2021.0008

43. Muecksch F, Wise H, Batchelor B, Squires M, Semple E, Richardson C, et al. Longitudinal Serological Analysis and Neutralizing Antibody Levels in Coronavirus Disease 2019 Convalescent Patients. J Infect Dis. 2021;223: 389–398. doi:10.1093/infdis/jiaa659

1. Castro MC, Kim S, Barberia L, Ribeiro AF, Gurzenda S, Ribeiro KB, et al. Spatiotemporal pattern of COVID-19 spread in Brazil. Science. 2021;372: 821–826. doi:10.1126/science.abh1558
2. WHO Health Emergency Dashboard. WHO Coronavirus (COVID-19) Dashboard. [cited 8 Nov 2022]. Available: https://covid19.who.int
3. WHO Health Emergency Dashboard. Brazil: WHO Coronavirus Disease (COVID-19) Dashboard With Vaccination Data. [cited 8 Nov 2022]. Available: https://covid19.who.int/region/amro/country/br
4. Worldometer. Brazil Population (2022). [cited 8 Nov 2022]. Available: https://www.worldometers.info/world-population/brazil-population/
5. Góes C, Karpowicz I. Inequality in Brazil: A Regional Perspective. 2017;2017: 34. doi:https://doi.org/10.5089/9781484324776.001
6. IBGE. Instituto Brasileiro de Geografia e Estatística. Projeções da População IBGE. [cited 7 Nov 2022]. Available: https://www.ibge.gov.br/estatisticas/sociais/populacao/9109-projecao-da-populacao.html?=&t=resultados
7. Secretaria Municipal da Saúde. Prefeitura da Cidade de São Paulo. Boletim Semanal Covid-19 no município de São Paulo de 14 de setembro de 2022. Prefeitura da Cidade de São Paulo; 14 Sep 2022 [cited 21 Dec 2022]. Available:

Formatted: Portuguese (Brazil)

Field Code Changed

Formatted: Portuguese (Brazil)

Formatted: Portuguese (Brazil)

Formatted: Portuguese (Brazil)

Field Code Changed

Formatted: Portuguese (Brazil)

https://www.prefeitura.sp.gov.br/cidade/secretarias/saude/vigilancia_em_saude/doencas_e_agravos/coronavirus/index.php?p=335024

8. Bryant JE, Azman AS, Ferrari MJ, Arnold BF, Boni MF, Boum Y, et al. Serology for SARS-CoV-2: Apprehensions, opportunities, and the path forward. Sci Immunol. 2020;5: eabc6347. doi:10.1126/sciimmunol.abc6347

9. Murhekar MV, Bhatnagar T, Selvaraju S, Saravanakumar V, Thangaraj JWV, Shah N, et al. SARS-CoV-2 antibody seroprevalence in India, August–September, 2020: findings from the second nationwide household serosurvey. Lancet Glob Health. 2021;9: e257–e266. doi:10.1016/S2214-109X(20)30544-1

10. Bergeri I, Whelan MG, Ware H, Subissi L, Nardone A, Lewis HC, et al. Global SARS-CoV-2 seroprevalence from January 2020 to April 2022: A systematic review and meta-analysis of standardized population-based studies. PLOS Med. 2022;19: e1004107. doi:10.1371/journal.pmed.1004107

11. Hallal PC, Hartwig FP, Horta BL, Silveira MF, Struchiner CJ, Vidaletti LP, et al. SARS-CoV-2 antibody prevalence in Brazil: results from two successive nationwide serological household surveys. Lancet Glob Health. 2020;8: e1390–e1398. doi:10.1016/S2214-109X(20)30387-9

12. Albuquerque JOM de, Kamioka GA, Madalosso G, Costa SA, Ferreira PB, Pino FA, et al. Prevalence evolution of SARS-CoV-2 infection in the city of São Paulo, 2020–2021. Rev Saúde Pública. 2021;55: 62–62. doi:10.11606/s1518-8787.2021055003970

Field Code Changed

Formatted: French (France)

Formatted: Portuguese (Brazil)

13. Tess BH, Granato CFH, Alves MCGP, Pintão MCT, Nunes MC, Rizzatti EG, et al. Assessment of initial SARS-CoV-2 seroprevalence in the most affected districts in the Municipality of São Paulo, Brazil. Braz J Infect Dis. 2021;25. doi:10.1016/j.bjid.2021.101604

14. FIOCRUZ. FIOCRUZ: Genomic Network. In: Genomahcov - Fiocruz [Internet]. [cited 9 Feb 2023]. Available: https://www.genomahcov.fiocruz.br/dashboard-pt/

15. WHO. Population-based age-stratified seroepidemiological investigation protocol for coronavirus 2019 (COVID-19) infection. World Health Organ. 2020; 20.

16. SEADE. Fundação Sistema Estadual de Análise de Dados. Sistema SEADE de Projeções Populacionais. [cited 7 Nov 2022]. Available: http://produtos.seade.gov.br/produtos/projpop/

17. IBGE. Instituto Brasileiro de Geografia e Estatística. Desigualdades sociais por cor ou raça no Brasil. [cited 20 Dec 2022]. Available: https://biblioteca.ibge.gov.br/index.php/biblioteca-catalogo?view=detalhes&id=2101972

18. Muench P, Jochum S, Wenderoth V, Ofenloch-Haehnle B, Hombach M, Strobl M, et al. Development and validation of the Elecsys Anti-SARS-CoV-2 immunoassay as a highly specific tool for determining past exposure to SARS-CoV-2. J Clin Microbiol. 2020;58.

19. Padoan A, Cosma C, Sciacovelli L, Faggian D, Plebani M. Analytical performances of a chemiluminescence immunoassay for SARS-CoV-2 IgM/IgG and antibody kinetics. Clin Chem Lab Med. 2020;58: 1081–1088. doi:10.1515/cclm-2020-0443

Formatted: Portuguese (Brazil)

Formatted: Portuguese (Brazil)

Formatted: Portuguese (Brazil)

Field Code Changed

Formatted: Portuguese (Brazil)

Formatted: Portuguese (Brazil)

Formatted: Portuguese (Brazil)

Field Code Changed

Formatted: Portuguese (Brazil)

20. Peluso MJ, Takahashi S, Hakim J, Kelly JD, Torres L, Iyer NS, et al. SARS-CoV-2 antibody magnitude and detectability are driven by disease severity, timing, and assay. Sci Adv. 2021;7: eabh3409. doi:10.1126/sciadv.abh3409
21. WHO. Interim statement on hybrid immunity and increasing population seroprevalence rates. 1 Jun 2022 [cited 21 Dec 2022]. Available: https://www.who.int/news/item/01-06-2022-interim-statement-on-hybrid-immunity-and-increasing-population-seroprevalence-rates
22. Statistical Methodology and Applications Branch, Surveillance Research Program, National Cancer Institute. Joinpoint Regression Program. 2022. Available: https://surveillance.cancer.gov/joinpoint/
23. Kim HJ, Fay MP, Feuer EJ, Midthune DN. Permutation tests for joinpoint regression with applications to cancer rates. Stat Med. 2000;19: 335–351. doi:10.1002/(sici)1097-0258(20000215)19:3<335::aid-sim336>3.0.co;2-z
24. Metzger C, Leroy T, Bochnakian A, Jeulin H, Gegout-Petit A, Legrand K, et al. Seroprevalence and SARS-CoV-2 invasion in general populations: A scoping review over the first year of the pandemic. PLOS ONE. 2023;18: e0269104. doi:10.1371/journal.pone.0269104
25. Rostami A, Sepidarkish M, Fazlzadeh A, Mokdad AH, Sattarnezhad A, Esfandyari S, et al. Update on SARS-CoV-2 seroprevalence: regional and worldwide. Clin Microbiol Infect. 2021;27: 1762–1771. doi:10.1016/j.cmi.2021.09.019
26. Duarte N, Yanes-Lane M, Arora RK, Bobrovitz N, Liu M, Bego MG, et al. Adapting Serosurveys for the SARS-CoV-2 Vaccine Era. Open Forum Infect Dis. 2022;9: ofab632. doi:10.1093/ofid/ofab632

Formatted: French (France)

Formatted: Portuguese (Brazil)

27. Buss L, Prete CA, Whittaker C, Salomon T, Oikawa MK, Pereira RHM, et al. Predicting SARS-CoV-2 Variant Spread in a Completely Seropositive Population Using Semi-Quantitative Antibody Measurements in Blood Donors. Vaccines. 2022;10: 1437. doi:10.3390/vaccines10091437

28. Jones JM, Stone M, Sulaeman H, Fink RV, Dave H, Levy ME, et al. Estimated US Infection- and Vaccine-Induced SARS-CoV-2 Seroprevalence Based on Blood Donations, July 2020-May 2021. JAMA. 2021;326: 1400–1409. doi:10.1001/jama.2021.15161

29. Barros AJD, Victora CG, Menezes AMB, Horta BL, Barros FC, Hartwig FP, et al. Population-level seropositivity trend for SARS-Cov-2 in Rio Grande do Sul, Brazil. Rev Saúde Pública. 2021;55. doi:10.11606/s1518-8787.2021055004075

30. Ribeiro KB, Ribeiro AF, Veras MA de SM, de Castro MC. Social inequalities and COVID-19 mortality in the city of São Paulo, Brazil. Int J Epidemiol. 2021;50: 732–742. doi:10.1093/ije/dyab022

31. Li SL, Pereira RHM, Jr CAP, Zarebski AE, Emanuel L, Alves PJH, et al. Higher risk of death from COVID-19 in low-income and non-White populations of São Paulo, Brazil. BMJ Glob Health. 2021;6: e004959. doi:10.1136/bmjgh-2021-004959

32. Carvalho MS, Bastos LS, Fuller T, Cruz OG, Damasceno L, Calvet G, et al. Incidence of SARS-CoV-2 over four epidemic waves in a low-resource community in Rio de Janeiro, Brazil: A prospective cohort study. Lancet Reg Health – Am. 2022;12. doi:10.1016/j.lana.2022.100283

33. Chen X, Chen Z, Azman AS, Deng X, Sun R, Zhao Z, et al. Serological evidence of human infection with SARS-CoV-2: a systematic review and

Formatted: Portuguese (Brazil)

Formatted: Portuguese (Brazil)

Formatted: Portuguese (Brazil)

Field Code Changed

Formatted: Portuguese (Brazil)

Formatted: Portuguese (Brazil)

Formatted: Portuguese (Brazil)

Formatted: Portuguese (Brazil)

Field Code Changed

Formatted: Portuguese (Brazil)

Formatted: German (Germany)

meta-analysis. Lancet Glob Health. 2021;9: e598–e609. doi:10.1016/S2214-109X(21)00026-7

34. Barberia LG, Cantarelli LGR, Oliveira MLC de F, Moreira N de P, Rosa ISC. The effect of state-level social distancing policy stringency on mobility in the states of Brazil. Rev Adm Pública. 2021;55: 27–49. doi:10.1590/0034-761220200549

35. Shook-Sa BE, Boyce RM, Aiello AE. Estimation Without Representation: Early Severe Acute Respiratory Syndrome Coronavirus 2 Seroprevalence Studies and the Path Forward. J Infect Dis. 2020;222: 1086–1089. doi:10.1093/infdis/jiaa429

36. Office for National Statistics (ONS). Coronavirus (COVID-19) Infection Survey, antibody data, UK: 5 October 2022. ONS website; 2022 Oct. Available: https://www.ons.gov.uk/peoplepopulationandcommunity/healthandsocialcare/conditionsanddiseases/bulletins/coronaviruscovid19infectionsurveyantibodyandvaccinationdatafortheuk/5october2022

37. National Institutes of Health. NIH begins study to quantify undetected cases of coronavirus infection. In: National Institutes of Health (NIH) [Internet]. 10 Apr 2020 [cited 7 Nov 2022]. Available: https://www.nih.gov/news-events/news-releases/nih-begins-study-quantify-undetected-cases-coronavirus-infection

38. Anderson RM, Vegvari C, Hollingsworth TD, Pi L, Maddren R, Ng CW, et al. The SARS-CoV-2 pandemic: remaining uncertainties in our understanding of the epidemiology and transmission dynamics of the virus,

Formatted: Portuguese (Brazil)

Formatted: French (France)

Formatted: French (France)

and challenges to be overcome. Interface Focus. 11: 20210008. doi:10.1098/rsfs.2021.0008

Supporting information

S1 Table. Frequency of selected characteristics of the sample population per survey, The SoroEpi MSP Study, Municipality of São Paulo, SP, Brazil, June 2020 to April 2022.

S2 Table. Frequency of adults (and 95% Confidence Interval) by type of anti-SARS-CoV-2 vaccine receivedgot and age group in Surveys 5, 6 and 7, The SoroEpi MSP Study, Municipality of São Paulo, SP, Brazil, June 2020 to April 2022.

S1 Data

Response to the academic editor and the reviewer

Manuscript Number PONE-D-23-14879

We thank both the academic editor and the reviewer for their valuable comments/suggestions. We have revised the entire text according to those comments as presented below.

Journal Requirements:

1. Please ensure that your manuscript meets PLOS ONE's style requirements, including those for file naming.

Response: We have adjusted our submitting materials to meet the PLOS ONE style templates.

2. Please include your updated Competing Interests statement in your cover letter; we will change the online submission form on your behalf.

Response: We have included the updated Competing Interests statement in our cover letter.

3. Please include your full ethics statement in the 'Methods' section of your manuscript file. In your statement, please include the full name of the IRB or ethics committee who approved or waived your study, as well as whether or not you obtained informed written or verbal consent. If consent was waived for your study, please include this information in your statement as well.

Response: We have included the information requested by the academic editor in the text (lines 174-178). Please refer to line 191 of the revised manuscript where we stated that "After participants signed the written informed consent, face-to-face interviews were conducted….".

4. We note that you have stated that you will provide repository information for your data at acceptance. Should your manuscript be accepted for publication, we will hold it until you provide the relevant accession numbers or DOIs necessary to access your data. If you wish to make changes to your Data Availability statement, please describe these changes in your cover letter and we will update your Data Availability statement to reflect the information you provide.

Response: Our dataset can be accessed at the University of São Paulo repository: https://sites.usp.br/epi-di/pagina-1/pagina-1-1/

Editor’s and reviewer’s comments:

5. The authors should consider several important studies that have looked at (pre-vaccination) SARS-CoV-2 antibodies in Sao Paulo. Namely, the authors need to compare their findings to the studies from Buss et al Science 2021 and Prete et al. eLife 2022, which looked at SARS-CoV-2 antibodies in (more cost effective) blood donor populations in Sao Paulo city using Abbot assays.

Response: We have included both references in the text (lines 382 and 390, references number 26 and 27).

6. Another important limitation is the lack of adjustments regarding sensitivity and specificity and seroreversion (if any) for the Roche assay.

Response: We have added in the text (lines 214-216) the 95%CI for sensitivity (99.5% [95% CI: 97.0-100.0]) and specificity (99.8% [95% CI: 99.7-99.9]) according to the development and validation of the Elecsys Anti-SARS-CoV-2 Immunoassay (Muench et al, 2020). Considering these parameters, the adjustment in prevalence would not produce statistically significant difference in results. In addition, Roche Elecsys is less prone to antibody waning (Muecksch 2021). Furthermore, the seroprevalence trend was not affected since we applied the same assays throughout study period. The study design consisted of seven independent cross-sectional population-based surveys that does not allow to measure antibody waning. Nevertheless, we considered these issues in the discussion section (lines 481-484).

Minor edits:

7. Results section could be restructured to present the survey results in chronological order (e..g reorder lines 282-288).

Response: We have made the changes to follow the chronological order.

8. Table 2, interpretation would be made easier if all categories were grouped Jun 2020 - Jan 2021; and Jan 2021 - Apr 2022.

Response: The time series analysis used in the present study presents parameters and statistical results of the JoinPoint models for each variable. These models identified the number of turning points and the period (trend segments) for each variable. The results of these analyses were able to show distinct growth trends for different categories. In fact, the JointPoint analysis allows us to show changes in the seroprevalence trend. Grouping or fixing the periods is not suitable for time series analysis. For example, the growth model showed no turning point and only one period (straight line for the entire period Jun 2020 - Apr 2022) for the high-income strata. In contrast, the model identified one turning point for the low-income strata and two trend periods (June 2020-October 2020; October 2020-April 2022). In order to make the interpretation easier, we modified the statistical analysis (lines 246-258) and the included a footnote for period in Table 2 (lines 343-344).

9. Lines 84-85, Sao Paulo was perhaps the most important site of entry of SARS-CoV-2 in the country in the early phase of the pandemic as the authors mention. This has been best shown in Candido et al. JTM 2020 and Candido et al. Science 2021.

Response: We have included the two studies by Candido et al in the Introduction section (references number 7 and 8, line 85).

10. - Lines 367-369. Include 95% CIs.

Response: We have included the 95% CI in line 369.

11. Lines 71. Compare and discuss results obtained from seroprevalence studies from Sao Paulo, including Buss et al. Science 2021 and Prete et al. eLife 2022.

Response: We have included a paragraph (lines 380-390, references number 26 and 27) in the Discussion section as suggested.
