# Supplementary material for: Seroprevalence trends of anti-SARS-CoV-2 antibodies in the adult population of the São Paulo Municipality, Brazil: Results from seven serosurveys from June 2020 to April 2022. The SoroEpi MSP Study
Source: PLoS One. 2024 Aug 26;19(8):e0309441. doi: 10.1371/journal.pone.0309441 (PMC11346932; doi:10.1371/journal.pone.0309441)
Supplement: Attachment — Submitted filename: Manusc 23-14879 Response to reviewer.docx [file pone.0309441.s003.docx]

Response to the academic editor and the reviewer

Manuscript Number PONE-D-23-14879

**We thank both the academic editor and the reviewer for their valuable comments/suggestions. We have revised** **the entire text according to those comments as presented below.**

Journal Requirements:

1. Please ensure that your manuscript meets PLOS ONE's style requirements, including those for file naming.

Response: We have adjusted our submitting materials to meet the PLOS ONE style templates.

2. Please include your updated Competing Interests statement in your cover letter; we will change the online submission form on your behalf.

Response: We have included the updated Competing Interests statement in our cover letter.

3. Please include your full ethics statement in the ‘Methods’ section of your manuscript file. In your statement, please include the full name of the IRB or ethics committee who approved or waived your study, as well as whether or not you obtained informed written or verbal consent. If consent was waived for your study, please include this information in your statement as well.

Response: We have included the information requested by the academic editor in the text (lines 174-178). Please refer to line 191 of the revised manuscript where we stated that “After participants signed the written informed consent, face-to-face interviews were conducted….”.

4. We note that you have stated that you will provide repository information for your data at acceptance. Should your manuscript be accepted for publication, we will hold it until you provide the relevant accession numbers or DOIs necessary to access your data. If you wish to make changes to your Data Availability statement, please describe these changes in your cover letter and we will update your Data Availability statement to reflect the information you provide.

Response: Our dataset can be accessed at the University of São Paulo repository: https://sites.usp.br/epi-di/pagina-1/pagina-1-1/

Editor’s and reviewer’s comments:

5. The authors should consider several important studies that have looked at (pre-vaccination) SARS-CoV-2 antibodies in Sao Paulo. Namely, the authors need to compare their findings to the studies from Buss et al Science 2021 and Prete et al. eLife 2022, which looked at SARS-CoV-2 antibodies in (more cost effective) blood donor populations in Sao Paulo city using Abbot assays.

Response: We have included both references in the text (lines 382 and 390, references number 26 and 27).

6. Another important limitation is the lack of adjustments regarding sensitivity and specificity and seroreversion (if any) for the Roche assay.

Response: We have added in the text (lines 214-216) the 95%CI for sensitivity (99.5% [95% CI: 97.0-100.0]) and specificity (99.8% [95% CI: 99.7-99.9]) according to the development and validation of the Elecsys Anti-SARS-CoV-2 Immunoassay (Muench et al, 2020). Considering these parameters, the adjustment in prevalence would not produce statistically significant difference in results. In addition, Roche Elecsys is less prone to antibody waning (Muecksch 2021). Furthermore, the seroprevalence trend was not affected since we applied the same assays throughout study period. The study design consisted of seven independent cross-sectional population-based surveys that does not allow to measure antibody waning. Nevertheless, we considered these issues in the discussion section (lines 481-484).

**Minor edits:**

7. Results section could be restructured to present the survey results in chronological order (e..g reorder lines 282-288).

Response: We have made the changes to follow the chronological order.

8. Table 2, interpretation would be made easier if all categories were grouped Jun 2020 - Jan 2021; and Jan 2021 - Apr 2022.

Response: The time series analysis used in the present study presents parameters and statistical results of the JoinPoint models for each variable. These models identified the number of turning points and the period (trend segments) for each variable. The results of these analyses were able to show distinct growth trends for different categories. In fact, the JointPoint analysis allows us to show changes in the seroprevalence trend. Grouping or fixing the periods is not suitable for time series analysis. For example, the growth model showed no turning point and only one period (straight line for the entire period Jun 2020 - Apr 2022) for the high-income strata. In contrast, the model identified one turning point for the low-income strata and two trend periods (June 2020-October 2020; October 2020-April 2022). In order to make the interpretation easier, we modified the statistical analysis (lines 246-258) and the included a footnote for period in Table 2 (lines 343-344).

9. Lines 84-85, Sao Paulo was perhaps the most important site of entry of SARS-CoV-2 in the country in the early phase of the pandemic as the authors mention. This has been best shown in Candido et al. JTM 2020 and Candido et al. Science 2021.

Response: We have included the two studies by Candido et al in the Introduction section (references number 7 and 8, line 85).

10. - Lines 367-369. Include 95% CIs.

Response: We have included the 95% CI in line 369.

11. Lines 71. Compare and discuss results obtained from seroprevalence studies from Sao Paulo, including Buss et al. Science 2021 and Prete et al. eLife 2022.

Response: We have included a paragraph (lines 380-390, references number 26 and 27) in the Discussion section as suggested.
