# Supplementary material for: Seroprevalence trends of anti-SARS-CoV-2 antibodies in the adult population of the São Paulo Municipality, Brazil: Results from seven serosurveys from June 2020 to April 2022. The SoroEpi MSP Study
Source: PLoS One. 2024 Aug 26;19(8):e0309441. doi: 10.1371/journal.pone.0309441 (PMC11346932; doi:10.1371/journal.pone.0309441)
Supplement: Attachment — Submitted filename: PONE-D-23-14879R2_Response to reviewer .docx [file pone.0309441.s005.docx]

**Response to the academic editor and the reviewers**

**Manuscript Number PONE-D-23-14879R2**

We thank the reviewers for their comments/suggestions. We have numbered the comments by Reviewer #2 following the order they were included in the body of the R1 manuscript. All comments were answered as presented below. We revised the entire text according to these comments.

**Note to Reviewer #2:** The authors are grateful for the time and effort you dedicated to reviewing our manuscript. Your detailed feedback has been invaluable in improving the quality and clarity of the paper. We noticed that you reviewed the manuscript by the end of December 2023, according to the dates recorded in all boxes containing your comments. We only got your comments on June 3, 2024. Unfortunately, the submission process and handle of our manuscript by the journal was troublesome and slow.

**ABSTRACT**

Note: We made small alterations in the abstract text to keep it within 300 words.

**Comment 1:** Line 50_R1: Add "in the pre-vaccination period".

**Response:** We have added the term as requested (Line 48_R2).

**Comment 2:** Line 55_R1: In comparison to whom? In comparison to what?

**Response:** We have rewritten part of the results section according to the reviewer´s comment (Lines 53-55_R2).

**INTRODUCTION**

**Comment 3:** Line 85_R1: It is not São Paulo route of viral spread. Please replace with the population of this city.

**Response:** We have rewritten the sentence in accordance with the reviewer’s request (Line 81_R2).

**Comment 4:** Line 111_R1: Specify the time period (before January 17, 2021.)

**Response:** We have specified the period as requested (Line 108_R2).

**METHODS**

**Comment 5:** Line 121_R1: Do you mean "9-20 September 2021" instead of "9-10 September 2021"?

**Response:** We have corrected the typo (Line 116_R2).

**Comment 6:** Line 123_R1: Why/How were these specific seven snapshot periods chosen?

**Response:** The dates of the surveys were established according to the progress of the epidemic in São Paulo and to include the pre and post vaccination periods (Lines 116-118_R2).

**Comment 7:** Line 141_R1: Add to Fig 2. data regarding the dominant variants of SARS-CoV-2 in accordance with the periods of their occurrence. As an example, I provide the reference below: Banjac J, Vuković V, Pustahija T, Bohucki N, Kovačević Berić D, Medić S, Petrović V, Ristić M. Epidemiological Characteristics of COVID-19 During Seven Consecutive Epidemiological Waves (2020-2022) in the North Bačka District, Serbia. Viruses. 2023;15, 2221.

**Response:** We have changed the title of Fig. 2 to “Timeline of COVID-19 cases and deaths, SARS-CoV-2 variants according to serosurveys, São Paulo, Brazil, 2020-2022” (Lines 139 and 140_R2).

We have included the predominant circulating variants in the footnote of Fig 2 as requested (Lines 141-143_R2).

**Comment 8:** Line 162_R1: If the census was conducted after 2010 but before the completion of the seventh study snapshot, it may compromise the results of this research. When was the last population census in Brazil, or specifically in São Paulo?

**Response:** Brazil's last population census was conducted in 2022 instead of 2020, and data were available in 2023. This delay was due to the COVID-19 pandemic and political constrains. The sizes of the census tracts were updated at each field work and weights were introduced to deal with this issue.

**Comment 9:** Line 178_R1: How is it possible that the study commenced six months prior to obtaining ethical committee approval for its implementation?

**Response:** We have made it clearer in the text. The original protocol was approved on May 15, 2020. January 14, 2021, was the date when the protocol’s amendment was approved. (Lines 179-181_R2).

**Comment 10:** Line 201_R1: Have all four COVID vaccines been available since January 17, 2021 in São Paulo? Were all four vaccines available throughout the entire vaccination period in São Paulo?

**Response:** We have rewritten this part of the text to make it clearer. (Lines 204-210_R2).

In São Paulo State, four types of vaccines were used based on their availability and immunization policies. The first vaccines introduced in the early phase of the Brazilian COVID-19 campaign were Coronavac (Sinovac), an inactivated whole-virus vaccine, and Covishield (AstraZeneca). These were followed by Ad26.COV2.S (Janssen) and BNT162b2 (Pfizer-BioNTech) vaccines. However, not all vaccines were available throughout the vaccination campaign due to shortages and delays. We included the reference: Li SL, et al BMJ Open 2024;14:e076354. doi:10.1136/ bmjopen-2023-076354.

**Comment 11:** Line 220_R1: Please specify the values sensitivity and specificity of the "Maglumi assay"

**Response:** We have specified the values of the sensitivity and the specificity of the IgM and IgG Maglumi assays (Lines 226-227_R2).

**RESULTS**

We have rewritten the first paragraph of the results to make the text clearer (Lines 277-280_R2).

**Comment 12:** Line 274_R1: Add the percentage.

**Response**: The percentage was included in the text (Line 278_R2).

**Comment 13:** Line 275_R1: Add the percentage.

**Response**: The percentage was included in the text (Line 279_R2).

**Comment 14:** Line 276_R1: Add the percentage.

**Response**: The percentage was included in the text (Line 279_R2).

**Comment 15:** Line 276-7_R1: Delete this sentence.

**Response:** The sentence was deleted.

**Comment 16:** Line 297_R1: According to the results in Table 1, this is not correct. Seroprevalence in the lower-income stratum was 16.5%, and in the high-income stratum was 13% during Survey 2.

**Response:** We have included the word “statistically” to indicate that in all surveys there was a significant difference, except for Survey 2 which showed a p-value of 0.20 in the statistical test (Line 298_R2).

**Comment 17:** Line 299_R1: Add (≥ 60 years).

**Response:** We have added the term in the sentence (Line 302_R2).

**Comment 18:** Line 299_R1: In the Table 1 is 6.0-16.2.

**Response:** We have corrected the typo (Line 303_R2).

**Comment 19:** Line 301_R1: Add (18-39 years).

**Response:** We have added the term in the sentence (Line 304_R2).

**Comment 20:** Line 303_R1: And, in comparison to the Asian or Indigenous group?

**Response:** We could not make statistical inferences because the precision of the estimates was too low. We have added a sentence in the text to explain why we have not used the estimates of the Asian or Indigenous group in our analyses: The Asian or Indigenous group did not provide reliable estimates due to its small sample size and large 95% confidence intervals (Lines 307-308_R2).

**Comment 21:** Line 304_R1: Add “significantly”.

**Response:** We have rewritten this part of the text to make it clearer (Line 306_R2).

**Comment 22:** Line 304_R1: According to the results in Table 1, this is not correct. The population with 16 or more years of education had lower seroprevalence in comparison with others across all seven serosurveys.

**Response:** We corrected the text according to the reviewer’s comment (Lines 309_R2).

**Comment 23:** Line 305_R1: It is necessary to add information and interpretation results indicating that in the Asian or Indigenous group, seroprevalence values do not increase exponentially over time, between 1 and 7 survey (**9.7%, 8.5%, 11.8%, 9.3%**, 28.7%, 45.5%, and 80.4%).

**Response:** We understand that it would have been interesting to study the Asian or Indigenous group, but the numbers were too small to allow for comparisons and interpretation of the results. Please refer to the response to comment 20.

**Comment 24:** Line 312_R1: In S2 Table, for the age group 40-59 years in Survey 7, the total vaccinated count is 340, while in Table 1, for the same age group and Survey 7, it is 360. Please explain this.

**Response:** We have checked our calculations and there was a typo in S2 Table – the correct is 288 and not 268, therefore the correct number is 360. We have corrected it in the S2 Table.

**Comment 25:** Line 315_R1 – Self-reported race/skin color: Why do the sums of respondents according to race/skin color differ from the Overall respondents across Surveys?

**Response:** This is because self-reported race/skin color was a variable with missing data. We have added a footnote in Table 1 with the number of missing information in each survey (Line 326_R2).

**Comment 26:** Line 338_R1. Why is there no representation of the Asian or Indigenous population on Fig 3d?

**Response:** In comments 20 and 23, we have explained why Asian or Indigenous population was not presented in the modeling analysis.

**DISCUSSION**

**Comment 27:** Line 364_R1: The main limitation of the discussion is the comparison of the results of this study with the results of seroprevalence studies on SARS-CoV-2 among blood donors. These are different categories with distinct methodological approaches.

**Response:** The second and third paragraphs of the discussion section have been rewritten to respond to the reviewer’s comment (Lines 380-409_R2).

**Comment 28:** Line 417_R1: It is necessary to explain the possible reasons for the seroprevalence of the Asian or Indigenous group, which did not exhibit exponential increases across Surveys 1 to 7= 9.7%, 8.5%, 11.8%, 9.3%, 28.7%, 45.5%, and 80.4%.

**Response:** Asian or Indigenous population were not included in this analysis because the prevalences could not be interpreted due to small sample size. Please refer to comments 20 and 23.

**Comment 29:** Line 432_R1: At the end (Survey 7), the seroprevalence values by age groups are as follows: 18-39 = 85.8%; 40-59 = 75.3%, and 60+ = 65.4%. Additionally, the percentage of unvaccinated individuals by age groups is: 18-39 = 2%; 40-59 = 2.6%, and 60+ = 0%. Are the lower seroprevalence rates of SARS-CoV-2 in the 60+ age group compared to younger categories a result of vaccination with different vaccines and/or less frequent infections with the SARS-CoV-2 virus in older individuals, or something else?

**Response:** We understand that the results mentioned in your comment refer to the last survey and are presented in Table 1 and S2 Table. These results were part of the descriptive analysis and not of the modeling analysis (Line 432_R1). In this regard, the interpretation of the increased growth of prevalence was based on time series analysis, specifically for the 60+ age group. We also showed the differences in the slopes among the three age groups during the study period (see Figure 3c). The high slope in prevalence growth occurred concurrently with the prioritization of COVID-19 vaccination in the older population. This last sentence is in the discussion section (Lines 439-441_R2).

Considering your comment, in the descriptive analysis, the lower prevalence of SARS-CoV-2 throughout the period suggests that older individuals were less exposed to the virus. Of note, the 60+ group was a priority group to start vaccination with CoronaVac as shown in the S2 Table. However, the serological marker applied in our survey does not allow us to distinguish between natural infection with SARS-CoV-2 and vaccine-induced immunity by different types of vaccines.

**Comment 30:** Line 469_R1: To dispel doubts regarding potential "bias" and "confounding" in the study design, it is necessary to explain the SARS-CoV-2 prevalence values that inversely correlate with the monitoring period.

**Response:** We have included two sentences in the limitations section of the discussion about the potential bias related to the low response rate in our study (Lines 482-484_R2).

**Comment 31:** Line 487_R1: Additionally, the study data do not indicate the duration that has passed since the moment of natural infection (primary or reinfection) and/or vaccination to the point of serum collection for testing antibodies against the SARS-CoV-2 virus.

**Response:** The time of natural infection or the vaccine intake in relation to the blood collection was out of the scope of our study. We have included one sentence in the limitation section of the discussion section to make it clearer (Lines 498-499_R2).
